# Delta SARS-CoV-2 s2m Structure, Dynamics, and Entropy: Consequences of the G15U Mutation

**DOI:** 10.1021/acsphyschemau.3c00008

**Published:** 2023-05-17

**Authors:** Joseph A. Makowski, Adam H. Kensinger, Caylee L. Cunningham, Caleb J. Frye, Morgan Shine, Patrick E. Lackey, Mihaela Rita Mihailescu, Jeffrey D. Evanseck

**Affiliations:** † Department of Chemistry and Biochemistry and Center for Computational Sciences, Duquesne University, Pittsburgh, Pennsylvania 15282, United States; ‡ Department of Biochemistry and Chemistry, Westminster College, New Wilmington, Pennsylvania 16172, United States

**Keywords:** COVID-19, structural bioinformatics, viral RNA, multivariate analysis, molecular dynamics

## Abstract

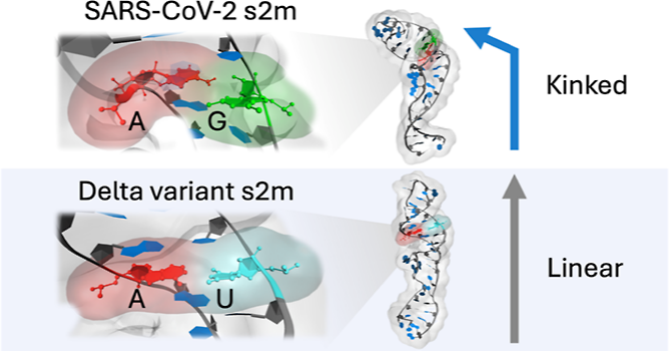

Bioinformatic analysis of the Delta SARS-CoV-2 genome reveals a single nucleotide mutation (G15U) in the stem-loop II motif (s2m) relative to ancestral SARS-CoV-2. Despite sequence similarity, unexpected differences between SARS-CoV-2 and Delta SARS-CoV-2 s2m homodimerization experiments require the discovery of unknown structural and thermodynamic changes necessary to rationalize the data. Using our reported SARS-CoV-2 s2m model, we induced the G15U substitution and performed 3.5 microseconds of unbiased molecular dynamics simulation at 283 and 310 K. The resultant Delta s2m adopted a secondary structure consistent with our reported NMR data, resulting in significant deviations in the tertiary structure and dynamics from our SARS-CoV-2 s2m model. First, we find differences in the overall three-dimensional structure, where the characteristic 90° L-shaped kink of the SARS-CoV-2 s2m did not form in the Delta s2m resulting in a “linear” hairpin with limited bending dynamics. Delta s2m helical parameters are calculated to align closely with A-form RNA, effectively eliminating a hinge point to form the L-shape kink by correcting an upper stem defect in SARS-CoV-2 induced by a noncanonical and dynamic G:A base pair. Ultimately, the shape difference rationalizes the migration differences in reported electrophoresis experiments. Second, increased fluctuation of the Delta s2m palindromic sequence, within the terminal loop, compared to SARS-CoV-2 s2m results in an estimated increase of entropy of 6.8 kcal/mol at 310 K relative to the SARS-CoV-2 s2m. The entropic difference offers a unique perspective on why the Delta s2m homodimerizes less spontaneously, forming fewer kissing dimers and extended duplexes compared to SARS-CoV-2. In this work, both the L-shape reduction and palindromic entropic penalty provides an explanation of our reported in vitro electrophoresis homodimerization results. Ultimately, the structural, dynamical, and entropic differences between the SARS-CoV-2 s2m and Delta s2m serve to establish a foundation for future studies of the s2m function in the viral lifecycle.

## Introduction

Mutations to severe acute respiratory syndrome coronavirus 2 (SARS-CoV-2), the virus responsible for COVID-19, have resulted in the emergence of many viral variants.^1^ Delta and Omicron SARS-CoV-2, each responsible for most of the COVID-19 cases in distinct phases of the pandemic, are characterized by “immune escape”, thwarting many public health strategies.^2−6^ While Delta SARS-CoV-2 has been replaced by less severe Omicron SARS-CoV-2 sublineages as the dominant variant worldwide,^7,8^ recent wastewater studies show that Delta SARS-CoV-2 remains in cryptic circulation^9^ and sequences continue to be deposited in the GISAID database (accession EPI_SET_230228yv).^10^ Considering the reported viral recombination between Delta and Omicron SARS-CoV-2, it remains imperative to understand the features of each variant to address future outbreaks swiftly.^11^ Because conserved genomic elements represent relatively static targets for antiviral intervention that are less prone to a mutation-driven immune escape,^6,12,13^ our interest is in understanding the functional role of the stem-loop II motif (s2m), a 41 nucleotide element conserved in coronaviruses prior to SARS-CoV-2.^12,14,15^

We previously reported differences in the atomistic structures and dynamics of the s2m of ancestral SARS-CoV and SARS-CoV-2.^16^ Despite only two mutations (U5C and G31U) distinguishing SARS-CoV-2 s2m from SARS-CoV s2m, experimental and computational evidence show that the secondary and tertiary structures change drastically due to a base pair register shift.^12,13,16,17^ Aside from U5C and G31U, it has been reported that SARS-CoV-2 s2m has obtained other mutations, including a G15U (G29742U) substitution, which was first reported in May 2020, and most recently in April 2022 within the “Deltamicron” recombinant lineage.^11,18−22^ In this case, Delta 21J_AY.4 and Omicron 21K/BA.1 variant genomes recombined resulting in a hybrid displaying signature mutations from both Delta and Omicron, with G15U being characteristic of the parent Delta variant.^11,23^

**Figure 1:**
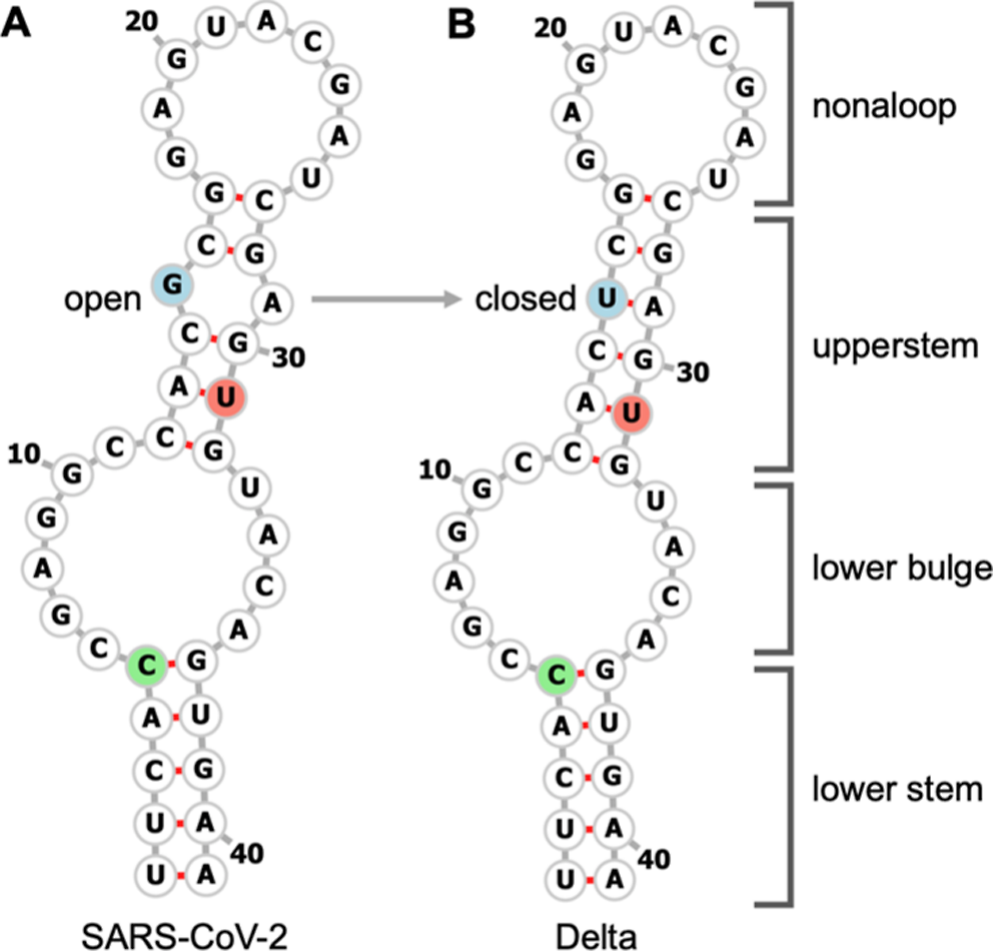
H^1^,H^1^-NOESY base pair assignments reported for the s2m in (A) SARS-CoV-2^17^ and (B) Delta SARS-CoV-2.^23^ Relative to SARS-CoV s2m, we have the U5C mutation in green, G31U in red, and G15U in blue.

Prior to the elucidation of the secondary structural differences between SARS-CoV and SARS-CoV-2 s2m by NMR spectroscopy assignments,^17^ and assuming homology between the two viruses, bioinformatics studies predicted that the SARS-CoV-2 G15U mutation would structurally disrupt the highly rigid GC-quartet s2m motif previously reported for SARS-CoV.^12,20,21^ Others have reported a 180 ns molecular dynamics (MD) simulation using the SARS-CoV s2m crystal structure (PDB: 1XJR) modified with the G15U mutation that exhibited structural destabilization of local base pairing and overall dynamics.^20^ However, our bioinformatics show that the G15U mutation exists in nearly all Delta SARS-CoV-2 s2m and instead results in a closed canonical Watson–Crick (WC) base pair in the upper stem, as revealed by NMR spectroscopy (Figure 1).^17,23^

We have reported the ability of SARS-CoV and SARS-CoV-2 s2m to form an extended duplex conformation mediated by a kissing complex intermediate, and our homodimerization experiments show the additional G15U mutation in Delta SARS-CoV-2 s2m results in further kinetic differences in the formation of the kissing complex and extended duplex relative to both SARS-CoV and SARS-CoV-2 s2m.^13,23^ Given our previous success in providing physical context and validation to our homodimerization experiments, we presently aim to expand our knowledge to include the Delta s2m.

In our current study, we elucidate a hierarchy of structural and dynamical features and thermodynamic parameters of the Delta s2m. To better understand and contextualize the structure and dynamics of the Delta s2m, we estimate absolute entropy of each s2m based on MD simulation position covariances. Ultimately, our simulation work rationalizes in vitro differences between SARS-CoV-2 and Delta s2m.^13^ Our results expand on the atomistic structure and dynamics and provide key information pertaining to the formerly dominant Delta variant or future recombinant variants.^21^

## Methods

### Molecular Dynamics Simulations

For starting coordinates, we mutated our previously reported SARS-CoV-2 s2m model to match the Delta s2m sequence,^16^ which were derived from a knowledge-based extrapolation of Wacker et al. NMR NOE assignments of SARS-CoV-2 s2m.^17^ Our unbiased simulation adopted a secondary structure consistent with our own Delta s2m NMR assignments,^23^ allowing us to make meaningful comparisons with our other experimental data. We prepared the Delta s2m for simulation analogously to our SARS-CoV and SARS-CoV-2 s2m models.^16^ tLeap from AmberTools20 was employed to create a TIP3P^24^ water model solvation box with 15 Å of padding from the solute, which is large enough to prevent self-interaction under periodic boundary conditions (Figure S1). The net charge of the system was neutralized by the addition of one Mg^2+^ cation and 39 Na^+^ atoms. The resulting concentration of [Mg^2+^] is approximately 3.5 mM, near the lower range of our experimental PAGE conditions. We simulated the system separately at 283 and 310 K, consistent with our NMR and PAGE experiments, respectively. The system was subject to 1000 steps of conjugate gradient energy minimization, followed by equilibration under a *NPT* ensemble^25^ until potential energy and volume had stabilized. Production run simulations were carried out for 3.5 μs. All simulations employed the AMBER^26^ force field with the *ff*99χOL3^27,28^ parameter set through the NAMD^29^ molecular dynamics engine for both RNA and ions.

Although the addition of one Mg^2+^ results in [Mg^2+^] within the range of our homodimerization experiments, we note that this likely underestimates Mg^2+^ condensation in the local ionic atmosphere of RNA (Figure S2). While intrinsic differences between the MD simulation and experiment, such as periodic boundary conditions, prevent complete agreement,^30−32^ our procedure is predicted to result in acceptably low error on the timescale of our simulations and provides a constant basis for comparison with our SARS-CoV s2m and SARS-CoV-2 s2m models.^16,31−33^

### Simulation Analysis

Routine 3D visualization and measurement of distances, angles, root-mean square deviation (RMSD), and root-mean square fluctuation (RMSF) were performed as implemented in visual molecular dynamics (VMD) software.^34^ Helical parameters were calculated with Web 3DNA 2.0 and visualized with the DSSR-PyMOL web application.^35,36^ Secondary structure cartoons were generated with ViennaRNA Forna Web Services^37^ or the Barnaba library for Python.^38,39^

### Multivariate Statistical Analysis

Molecular dynamics (MD) simulations yield high-dimensional “big data”, making detailed structural analysis intractable with traditional approaches alone. Principal component analysis (PCA) is a popular method for dimensionality reduction which projects data down to a subspace responsible for a large proportion of the total variance.^40^ This is facilitated by finding the eigenvectors of the covariance matrix of atomic coordinates **C**, where each respective eigenvalue is a proportion of the total position variance. Ultimately, this procedure enables classification of simulated structures into conformational substates (CS), organized by structural similarity and dissimilarity.^40,41^ Application of the *k*-means clustering algorithm partitioned PCA-reduced MD data into CSs that maximize dissimilarity between substates and identify representative centroid structures.^16,42^ We performed PCA on different selections of atoms, particularly, the entire s2m (nt. 1–41) and the terminal loop (nt. 17–27), to characterize both global and local dynamics suspected to be relevant for kissing complexation or extended duplexation. Because at least 50% of the total variance was captured within three principal components for all systems and selections, as portrayed by scree plots, we generally projected our simulation data to a 3D subspace and visualize by a combination of 2D projections (Figure S3).^43,44^ This was facilitated by an in-house Python script employing the libraries of MDTraj to parse simulation data,^45^ Scikit-learn to perform PCA and clustering,^46^ and Matplotlib for graphics.^47^

### Estimation of Entropy

Due to the computational expense of calculating absolute entropy explicitly in an *NPT* ensemble with correlated N-body dynamics,^48^ we previously reported estimations of s2m absolute entropy using the quasiharmonic approximation for macromolecular MD simulations.^49,50^ Under the assumption that fluctuations in atomic coordinates follow a multivariate Gaussian distribution, it is possible to use position variances from a mass-weighted covariance matrix to estimate absolute entropy. To compare entropic effects on different homodimerization processes, we calculated the entropy associated with the entire s2m, the terminal loop atoms (nt. 17–27), and the palindromic sequence (nt. 20–23). AMBER mass parameters were used to populate the mass matrix used for our calculations. Further details are provided in the Supporting Information.

## Results and Discussion

### Structure and Dynamics of the Delta s2m

#### Secondary Structure

To facilitate comparisons with our previous models, the initial coordinates of the Delta s2m simulation were almost identical to our previous SARS-CoV-2 s2m model, with one mutation at the fifteenth position (G15U). Despite previous challenges with homology modeling,^16^ simulations close the bulge through the U15-A29 base pair, at both temperatures. Yet, as discussed in greater detail below, the latter half of the 310 K simulation allowed for additional sampling of terminal loop conformations. In contrast, the system simulated at 283 K adopted and maintained a secondary structure consistent with our NMR assignments.^23^

#### s2m Dynamics

**Figure 2:**
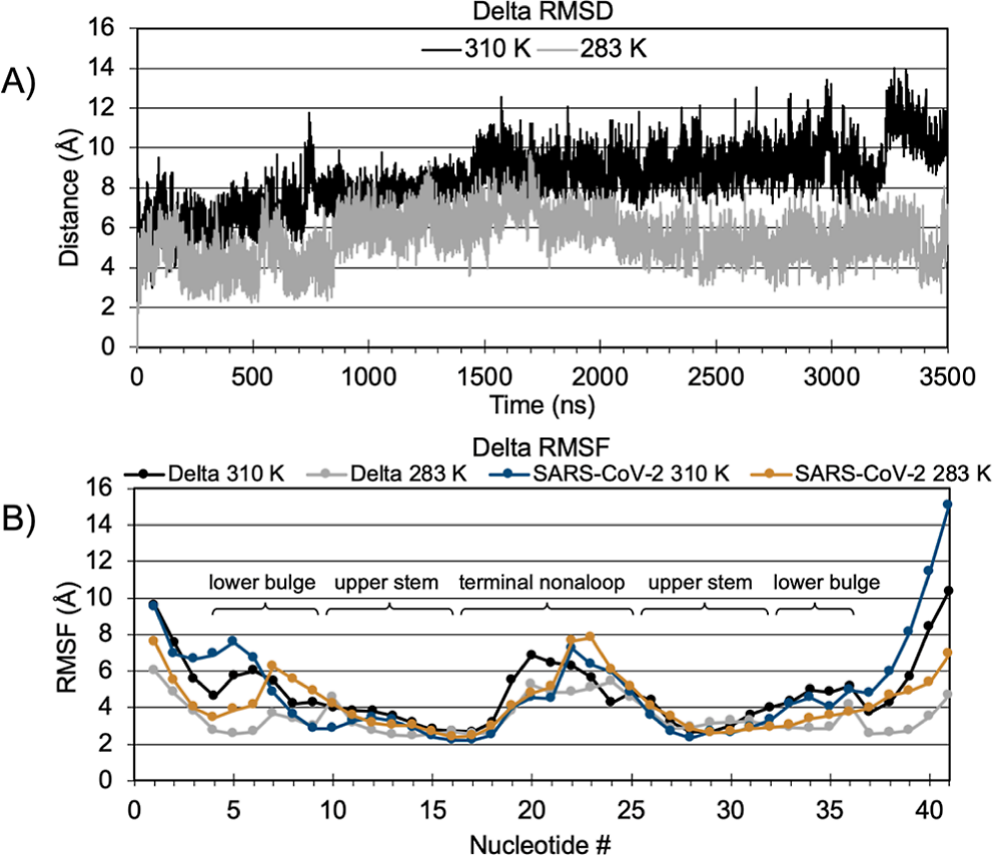
(A) Backbone RMSD of Delta s2m models. (B) Nucleotide heavy atom RMSF of Delta s2m models. The first frame of the simulation following equilibration was used as the reference structure.

The total structure had an average backbone heavy atom RMSD of 8.4 ± 1.5 Å at 310 K (Figure 2A). Following equilibration, the system deviation steadily increased for ca. 1.5 μs before stabilizing at an average of 9.3 ± 0.6 Å. During the last 200 ns, the deviation increased again to a peak of 14 Å. Upon visual inspection, this increase of RMSD is due to concerted fluctuations of the lower stem and slight fraying. RMSF of Delta s2m revealed comparable fluctuations to SARS-CoV-2 on a per nucleotide basis (Figure 2B). Again, the nucleotides with the highest fluctuation are the lower stem, including nucleotides 1–2 and 39–41 ranging from 5.5 to 11 Å due to fraying. The terminal nonaloop showed elevated fluctuations for nucleotides 19–26 ranging from 5.5 to 7.0 Å, containing the palindromic sequence of interest for homodimerization.

PCA over all the Delta s2m heavy atoms at 310 K reveals that fluctuations in RMSD are a consequence of large-magnitude tertiary movement of the lower stem (Figure S4), resulting in a single CS rather than multiple well-defined CSs (Figure S3). This is especially true for the 283 K simulation, which had only one isotropic cluster for whole-s2m heavy-atom PCA. In contrast with the SARS-CoV-2 s2m, there is not a significant amount of fraying; instead, the intact lower stem moves as a collective unit, “hinged” just below the lower bulge. The collective structure of the stem above the lower bulge remains mostly fixed, except for dynamics local to specific regions of the s2m (discussed below). Through the lens of extended duplex formation, these data suggest that Delta s2m is less prone to fraying and base melting than SARS-CoV-2, consistent with our experimental data.

#### Absence of L-Shape Kink

**Figure 3:**
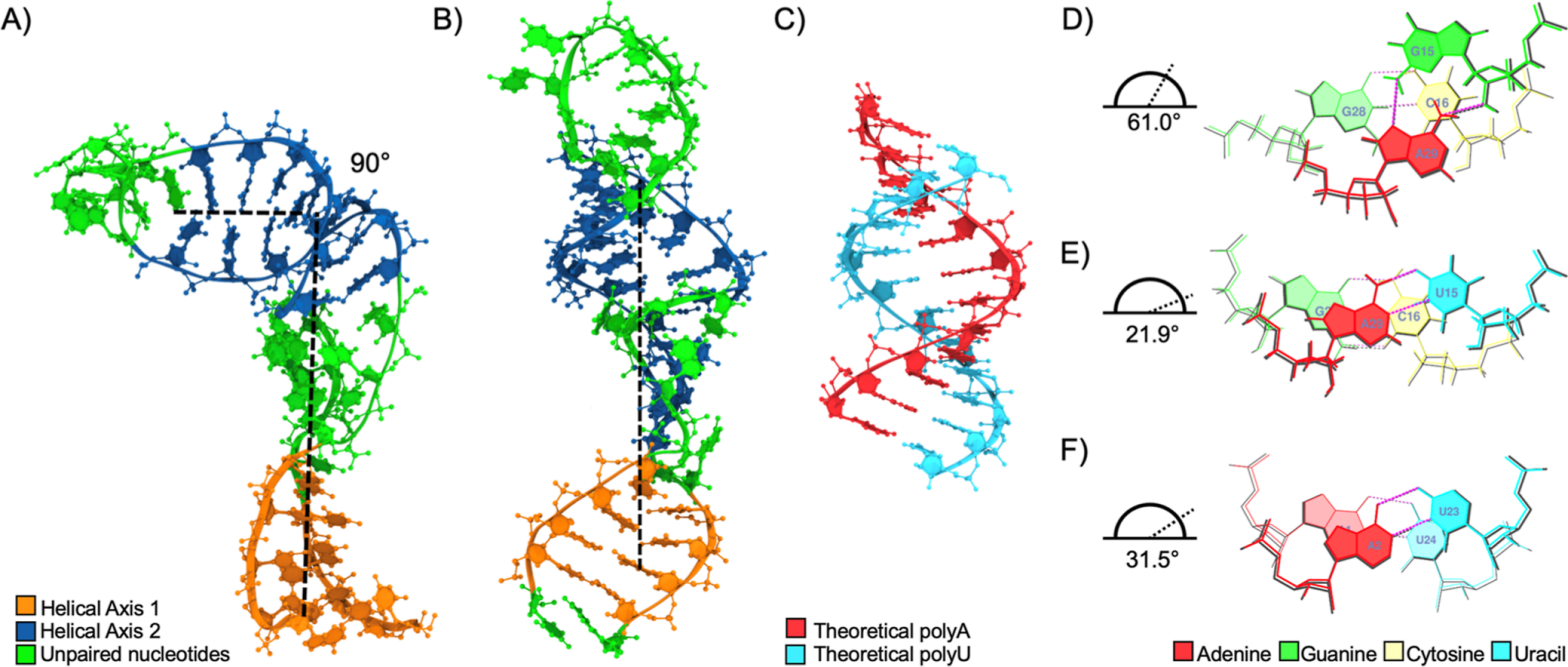
(A) L-shaped kink in the previously reported SARS-CoV-2 s2m model.^16^ (B) Absence of L-shaped kink in Delta s2m 310 K models. Black dashed lines indicate 90 and 180° angles between the two helical axes of SARS-CoV-2 and Delta s2m, respectively. (C) Theoretical poly(A)-poly(U) A-form RNA double helix with ideal geometric parameters for comparison to simulated models. (D) 15th position base step twist of SARS-CoV-2 terminal loop CS1, (E) 15th position base step twist of Delta terminal loop CS1 (see below), and (F) ideal A-form RNA base step twist for comparison. (A) Reproduced from Kensinger, A. H.; Makowski, J. A.; Pellegrene, K. A.; Imperatore, J. A.; Cunningham, C. L.; Frye, C. J.; Lackey, P. E.; Mihailescu, M. R.; Evanseck, J. D. Structural, Dynamical, and Entropic Differences between SARS-CoV and SARS-CoV-2 S2m Elements Using Molecular Dynamics Simulations. *ACS Physical Chemistry Au***2023, 3 (1),** 30–43. https://doi.org/10.1021/acsphyschemau.2c00032. Copyright 2023 American Chemical Society.

We previously reported an “L-shape kink” in the three-dimensional structure of SARS-CoV-2 s2m analogous to the tertiary kink observed in the SARS-CoV s2m crystal structure (Figure 3A).^12,16^ Notably, the Delta s2m shape deviates from both SARS-CoV and SARS-CoV-2 s2m by the absence of an L-shaped kink (Figure 3B). Using the Web 3DNA 2.0 webserver, helical parameters were calculated to define two helical axes consisting of the lower stem and the upper stem, interrupted by the unpaired nucleotides in the lower bulge. There is an apparent absence of a hinge point to form the L-shape kink; instead, Delta s2m forms a “linear” hairpin with greatly reduced bending dynamics in the upper stem. Helical characterization of the local base pair geometries included both individual base-step and helical reference frames.^36,52^ Each CS of the Delta s2m upper stem was classified as an A-form helix (Tables S1–S3). In contrast, we previously reported that the SARS-CoV-2 s2m upper stem helix included helical distortions at the 15th position noncanonical G:A base pair that strongly deviated from the ideal A-form RNA helix (Figure 3C) that are largely responsible for a kink in the hairpin (Table S4).^16^

At the 15th position, the SARS-CoV-2 s2m base-step twist was found to be 61.0°, which is nearly double the ideal A-form twist of 31.5° and triple the Delta twist of 21.9° (Figure 3D–F and Table S5). Compared to ideal A-form RNA and Delta s2m, the SARS-CoV-2 15th base step has lowered inclination and increased tip parameters, which contribute to the upper helical axis adopting a conformation nearly orthogonal to that of the lower stem (Table S6). Thus, the G15U mutation was found to correct the deformation to an unkinked helical shape by replacing the noncanonical G:A pair with the canonical A:U pair and restoring helical base stacking (Figures S5 and S6).

In contrast to SARS-CoV-2 s2m, no long-range interaction that could serve to stabilize the backbone for a hinge point for an L-shaped kink was found. This result rationalizes observed differences in migration patterns in PAGE experiments,^23^ where the Delta s2m monomer band is slightly more mobile than the SARS-CoV-2 s2m monomer, suggestive of a difference in shape.

#### Terminal Loop Dynamics

**Figure 4:**
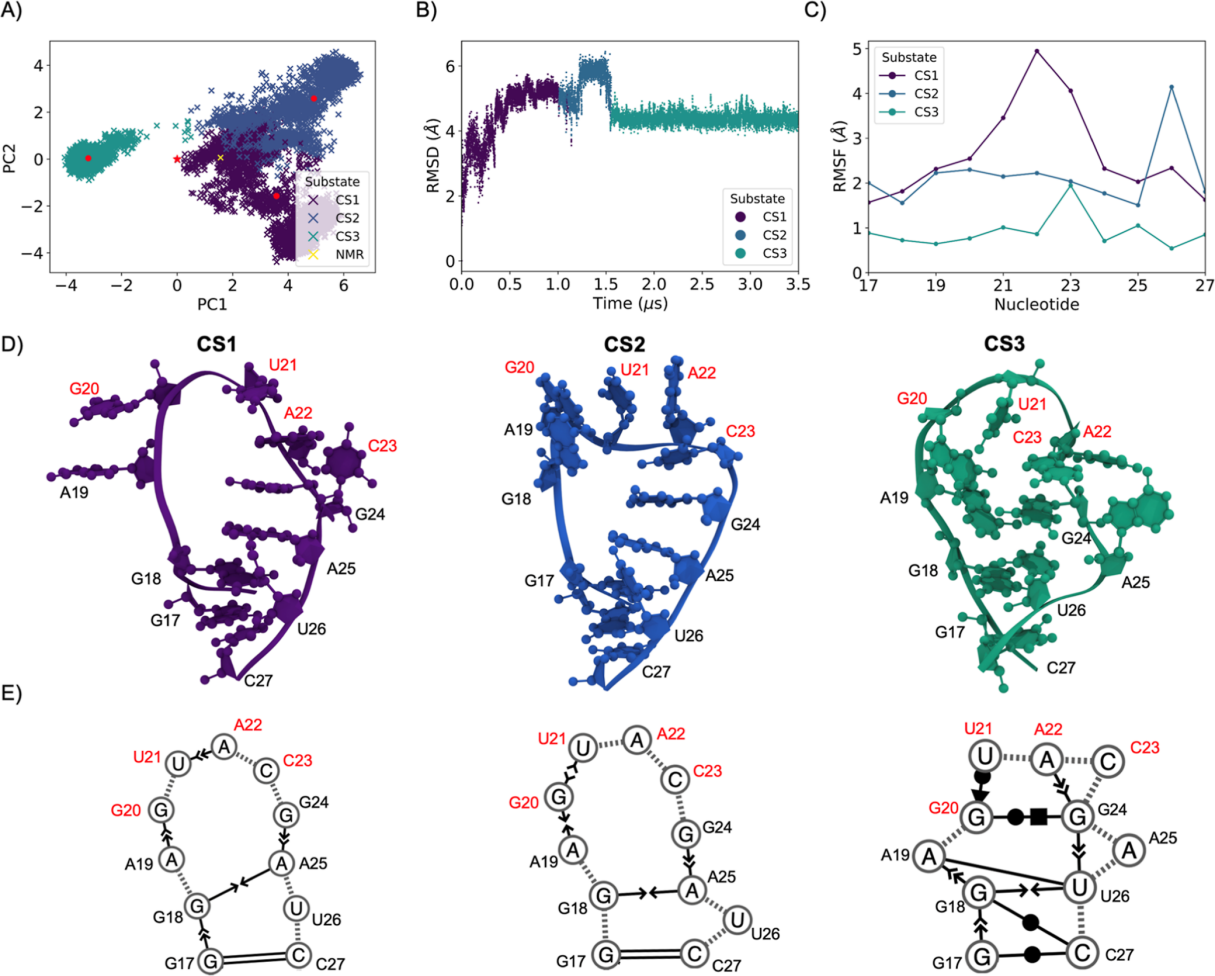
Multivariate analysis of the Delta SARS-CoV-2 s2m terminal loop at 310 K. (A) Three CSs were identified through terminal loop heavy-atom PCA. The PCA average structure is given by a red star, and the NMR-based starting coordinates by a yellow cross. (B) Terminal loop RMSD relative to the first frame post-equilibration colored by CS. (C) RMSF of structures within each CS relative to each centroid structure. (D) Centroid structures of each CS. Palindromic nucleotides are labeled in red. (E) Secondary structure and base stacking interactions determined through Barnaba. Dashed lines represent the backbone, oriented arrows base stacking interactions and base pairs in Leontis–Westhoff notation.^51^

Multivariate analysis of our 310 K simulation revealed three substates of the Delta nonaloop (Figure 4A). The basis of differences between each substate is the same as the SARS-CoV-2 nonaloop as in Delta fundamentally. In general, we find stack swapping or base pair melting dynamics transiently displace nucleotides, causing them to be highly solvent exposed and dynamic until further swapping results in a transition to a different conformational substate. As evidenced by identification of RMSD with each CS, the terminal loop begins the simulation perturbed, experiencing high fluctuations in RMSD and increased RMSF (Figure 4B,C). The terminal loop arrives at a relatively stable structure for CS3, for which RMSD remains nearly constant and PCA reveals a tight, isotropic cluster around the centroid, suggestive of low intra-CS structural variation. RMSF for CS1 and CS2 (approximately 2 Å) was generally found to be higher than the centroids of the SARS-CoV-2 terminal loop (approximately 1 Å); only Delta CS3 fluctuated comparably to the SARS-CoV-2 nonaloop. The difference between CS3 and the prior two CS is stark: previously free U26 swings back into a base stack with G18, and backbone folding results in a greater number of new stacking interactions and base pairs, as depicted by the Barnaba secondary structure. This plurality of stabilizing interactions rationalizes the rigid RMSD throughout CS3 (Figure 4D,E), where the loop remains trapped in a local minimum until the end of the simulation. Thus, despite fundamental similarities in base stacking, reshuffling, and melting, we find greater dynamics and entropy in the Delta s2m terminal loop, as described below.

CS1 of the 310 K simulation is characterized by highly dynamic palindromic nucleotides, as evidenced by the broad peak in RMSF from nucleotides 21–23, with additional stabilization conferred to G20 by its stack with A19. In CS3 of the 310 K simulation, a notable WC A19-U26 base pair formed and persisted until the end of the simulation. As one would expect by the conventional understanding of RNA dynamics,^53,54^ this pair was not detected in our H^1^, H^1^-NOSEY experiments performed at 283 K,^23^ yet we find CS3 is a local minimum due to the higher simulated temperature. Thus, to make more direct comparisons with NMR, we analyzed the terminal loop of our 283 K simulation with multivariate methods in an analogous manner.

**Figure 5:**
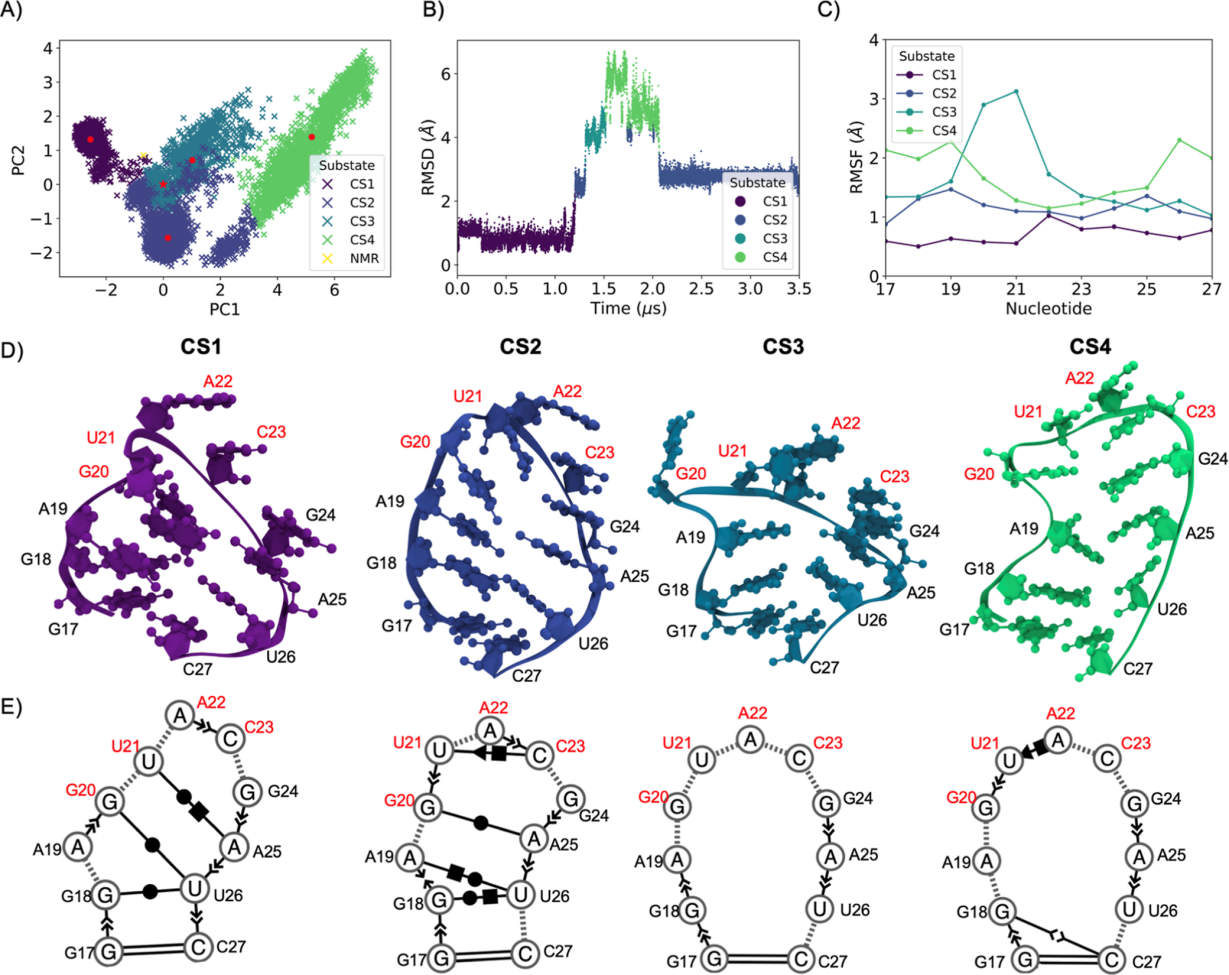
Multivariate analysis of the Delta SARS-CoV-2 s2m terminal loop at 283 K. (A) Four CSs were identified through terminal loop heavy atom PCA. The PCA average structure is given by a red star, and the NMR-based starting coordinates by a yellow cross. (B) Terminal loop RMSD relative to the first frame post-equilibration colored by CS. (C) RMSF of structures within each CS relative to each centroid structure. (D) Centroid structures of each CS. Palindromic nucleotides are labeled in red. (E) Secondary structure and base stacking interactions determined through Barnaba. Dashed lines represent the backbone, oriented arrows base stacking interactions, and base pairs in Leontis–Westhoff notation.^51^

Our simulation at 283 K resulted in similar dynamics and increased structural variability relative to 310 K (Figure 5). Through PCA, four CSs were resolved (Figure 5A). Both CS1 and CS2, which, respectively, represent the beginning and end of the simulation, are characterized by an increase in a multitude of stabilizing base stacking interactions and non-canonical base pairs. In CS1, this is reflected by low, nearly constant terminal loop RMSD and nucleotide RMSF (Figure 5B,C), with no apparent swung-out nucleotides. All nucleotides fluctuate more in CS2 and increase in deviation from the starting structure, but RMSD is also nearly constant, reflecting the abundance of stabilizing interactions. On the other hand, CS3 and CS4 represent a comparatively dynamic transition in the middle of the simulation (Figure 5B), with interactions breaking (Figure 5D,E) to produce an open terminal loop, palindromic nucleotides destabilized and not preorganized for kissing complexation. The transition to CS3 and CS4 is reflected by a peak in RMSD and increased nucleotide RMSF. In the transition from CS2 to CS3, stacking and noncanonical pairs involving G20 and U21 were broken, leaving each swung-out and unstacked, resulting in a prominent peak in RMSF relative to other nucleotides. However, the G20-U21 stack returns in CS4 and remains until the end of the simulation in the return to CS2. Overall, the Delta s2m terminal loop is characterized by high amounts of dynamic variability at either temperature, suggestive of an entropic penalty to homodimerization comparable to SARS-CoV-2 s2m.

#### Backbone Distortion

**Figure 6:**
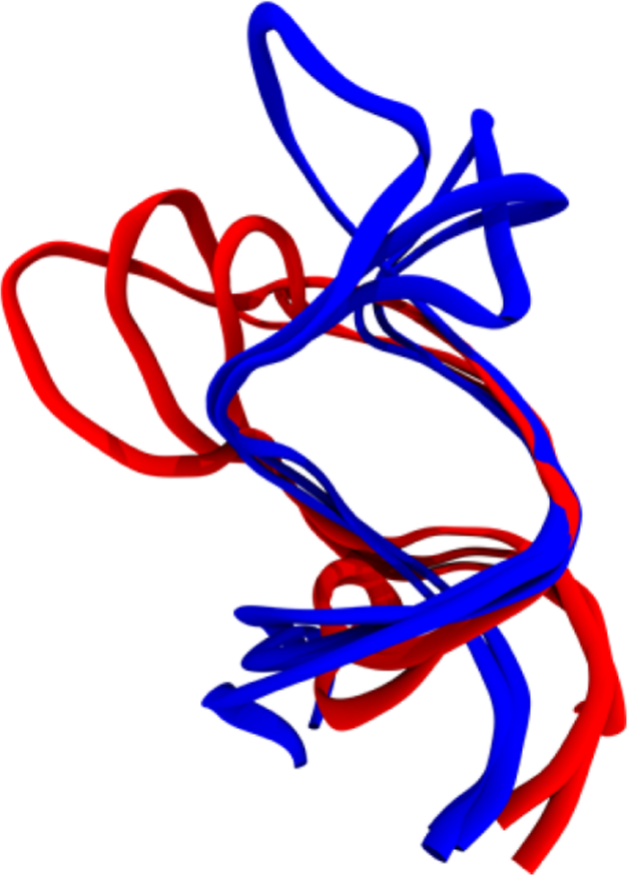
Terminal loop backbones of SARS-CoV-2 CS centroids (red) superimposed with Delta CS centroids (blue), aligned to nucleotide 15 and each from simulations at 310 K.

Although the same number of Tier-1 CS was determined by PCA and *k*-means for the 310 K simulations of SARS-CoV-2 s2m and Delta s2m, there exist key structural differences between the systems. First, in the SARS-CoV-2 s2m model, the noncanonical G15:A29 base pair within the upper stem introduced a stem defect due to the lower occupancy of the associated hydrogen bonds (55.5%). In contrast, the calculated occupancy for the canonical U15:A29 base pair in Delta was 80.9%. While the canonical base pair does have minor fluctuations, it is much more stable and does not exhibit the complete melting dynamics or transiently swing out to interact with solvent, as in SARS-CoV-2. A notable consequence attributed to this change is that every substate of the 310 K Delta nonaloop has a greater helical content than the SARS-CoV-2 s2m, which forms bent relatively parallel tracks in the nonaloop. Superposition of the Delta and SARS-CoV-2 centroid terminal loop backbones (Figure 6) underscores the differences in shape governed by the hairpin helicity. The 283 K simulation was similar in its deviation from the SARS-CoV-2 s2m terminal loop.

#### Tier 2 Dynamics

Repeated PCA over each Tier-1 CS resulted in the simulation being re-partitioned into new Tier-2 CS (Figure S7). The three Tier-1 terminal loop CSs of the 310 K simulation were partitioned into a total of 10 Tier-2 CSs, while the four 283 K terminal loop CSs contained a total of 12 Tier-2 CSs. In comparison, we previously reported that the SARS-CoV-2 terminal loop at 310 K sampled three Tier-1 CS and seven Tier-2 CS. Broadly, the Tier-2 substates are differentiated by a combination of stack swapping, base pair isomerization, and modes not dissimilar to U21 “up” and “down” reported for SARS-CoV s2m while retaining characteristic features of each Tier-1 CS. While in-depth analysis of Tier-2 is intractable for such highly dynamic systems, details pertaining to the Tier-2 PC spaces and centroid secondary structures are provided in the Supporting Information. Despite sharing the same sequence and starting from the same coordinates, the G15U mutation induced heightened dynamics and greater sampling of microstates within the Delta s2m terminal loop.

From both traditional and multivariate analyses, a comparison between SARS-CoV-2 and Delta variant s2m indicates that Delta is more flexible in the terminal loop sampling conformations with increased RMSF. Specifically, at 310 K, stack reshuffling and base swing-outs define the first two conformational substates before folding into a stable organization in CS3. Notably, CS1 features the palindromic nucleotides concertedly swung out and solvent exposed, and the 283 K simulation sampled similar dynamics without deviating from the NMR secondary structure assignments. Globally, the Delta s2m motion is dominated by swinging of the lower stem yet minimal fraying occurred to potentially increase the entropic penalty for extended duplexation. In contrast to SARS-CoV-2 which contains nonstandard helices, the upper stem in Delta s2m is classified as an A-form helix due to the canonical U15:A29 base pair. Thus, our structural and dynamical analyses suggest the Delta s2m is not preorganized for the formation of the kissing complex or extended duplex, consistent with observations from the reported experiments.^23^

### Entropy of the Delta s2m

Previously, we found that our model of the SARS-CoV-2 s2m was generally far more entropic than SARS-CoV s2m. This result was not difficult to rationalize considering experimental and computational evidence revealing dramatic changes in the secondary and tertiary structures of the s2m between the two viruses. Presently, we show that differences also exist between the SARS-CoV-2 and Delta s2m (Table 1).

**Table 1:** Estimated Relative Entropy of the s2m Using the Quasiharmonic Approximation^a^

oligomer	palindrome [kcal mol^–1^K^–1^]	terminal loop [kcal mol^–1^K^–1^]	entire s2m [kcal mol^–1^K^–1^]
SARS-CoV-2	0.000	0.000	0.026
Delta	0.022	0.005	0.000

a Based on data from 17,500 frames (3.5 μs) of MD simulation, systems simulated at 310 K. Palindrome: all atoms in nt. 20–23 were used. Terminal loop: all atoms in nt. 17–27 were used.

#### Terminal Loop Entropy

Compared to the difference between SARS-CoV and SARS-CoV-2, a relatively small difference exists between SARS-CoV-2 and Delta terminal loops, translating to an energetic contribution of approximately 1.8 kcal/mol at 310 K. We rationalize this similarity by noting that the SARS-CoV-2 and Delta s2m terminal loops adopt the same general nonaloop secondary structure. The estimated entropic difference is small enough that it may be a consequence of error in the quasiharmonic method or simulation sampling limitations and should not be over-interpreted. However, if we assume the difference is physically meaningful, then the increase in entropy correlates with our observation of reduced kissing dimer complexation in the Delta variant.

#### Palindromic Sequence Entropy

Previously, we found the SARS-CoV-2 s2m palindromic sequence was 0.045 kcal mol^–1^ K^–1^ higher in entropy than SARS-CoV s2m, translating to a free energy contribution of approximately 14 kcal/mol. Interestingly, between the two nonaloop palindromes, we find that the Delta s2m palindromic sequence is even higher in entropy, translating to an entropic free energy penalty of approximately 6.8 kcal/mol at 310 K relative to SARS-CoV-2 (approx. 21 kcal/mol relative to SARS-CoV s2m). This heighted penalty correlates with our homodimerization experiments which show no kissing complex formation in the Delta s2m and slight kissing complexation in SARS-CoV-2 s2m.

#### Entire s2m Entropy

Per our previous study, we expect the entropy of the entire s2m to reflect the rate of conversion to the extended duplex conformation. Of the three viruses studied, our model of the SARS-CoV-2 s2m remains highest in entropy. The Delta s2m is also much higher in entropy than the SARS-CoV s2m, with a predicted absolute entropy closer to SARS-CoV-2 s2m. However, as expected from less fraying and fewer high-magnitude tertiary modes, Delta s2m faces a greater penalty to extended duplexation than SARS-CoV-2 s2m. This correlates with the experimental results showing the Delta variant s2m forms a small amount of extended duplex more than SARS-CoV and less than SARS-CoV-2.

## Conclusions

Our simulations show meaningful structural, dynamical, and entropic differences arising from the G15U mutation in the Delta variant s2m. Addition of the G15U mutation to our previous SARS-CoV-2 s2m model results in several important changes in the tertiary structure, including an absence of an L-shaped kink and distortion of the upper-stem and terminal loop backbone relative to SARS-CoV-2 s2m. A simulated three-dimensional s2m shape offers an explanation for nonidentical hairpin migration in our reported electrophoresis experiments. Despite both having an identical number of bases, the linearly shaped Delta s2m is observed to have higher mobility in the gel medium than the kinked SARS-CoV-2 s2m, as expected. The dynamics sampled in the terminal nonaloop were fundamentally similar to SARS-CoV-2, but PCA revealed increased dynamics within each Delta s2m terminal loop CS, as well as a more varied dynamical hierarchy relative to SARS-CoV-2, suggestive of more accessible microstates. Employing the quasiharmonic approximation for entropy, the terminal loop and palindromic sequence of the Delta s2m was computed to have a higher entropy than SARS-CoV-2 s2m, suggesting that the Delta s2m homodimerizes less spontaneously. However, the entire Delta s2m was lower in entropy than SARS-CoV-2 s2m and experienced dramatically less fraying at both temperatures, suggesting that extended duplex formation is also less spontaneous for the Delta s2m, in alignment with our experimental observation that Delta s2m forms fewer kissing dimers and extended duplexes compared to SARS-CoV-2. Our work provides the foundation for future studies on the mechanism of homodimerization in the Delta variant, providing a basis for establishing structure–function connections. Ultimately, our study establishes the atomistic three-dimensional structure and uncovers dynamic differences that arise from a single s2m sequence change from SARS-CoV-2 to the Delta variant.

## Supporting Information Available

Structural and dynamical data, principal component analysis, simulation details, and details about the quasiharmonic estimation of absolute entropy (PDF)
