# Supplementary material for: Delta SARS-CoV-2 s2m Structure, Dynamics, and Entropy: Consequences of the G15U Mutation
Source: ACS Phys Chem Au. 2023 May 17;3(5):434–43. doi: 10.1021/acsphyschemau.3c00008 (PMC10540284; doi:10.1021/acsphyschemau.3c00008)
Supplement: Supplementary file 1 — pg3c00008_si_001.pdf [file pg3c00008_si_001.pdf]

Supporting Information

# Delta SARS-CoV-2 s2m Structure, Dynamics, and Entropy: Consequences of the G15U Mutation

Joseph A. Makowski[‡1], Adam H. Kensinger[‡1], Caylee L. Cunningham[1], Caleb J. Frye[1], Morgan Shine[§2], Patrick E. Lackey[2], Mihaela Rita Mihailescu[1], and Jeffrey D. Evanseck[*1]

[1]Department of Chemistry and Biochemistry and Center for Computational Sciences, Duquesne University, Pittsburgh, PA, 15282, USA

[2]Department of Biochemistry and Chemistry, Westminster College, New Wilmington, PA, 16172, USA

*Email: evanseck@duq.edu

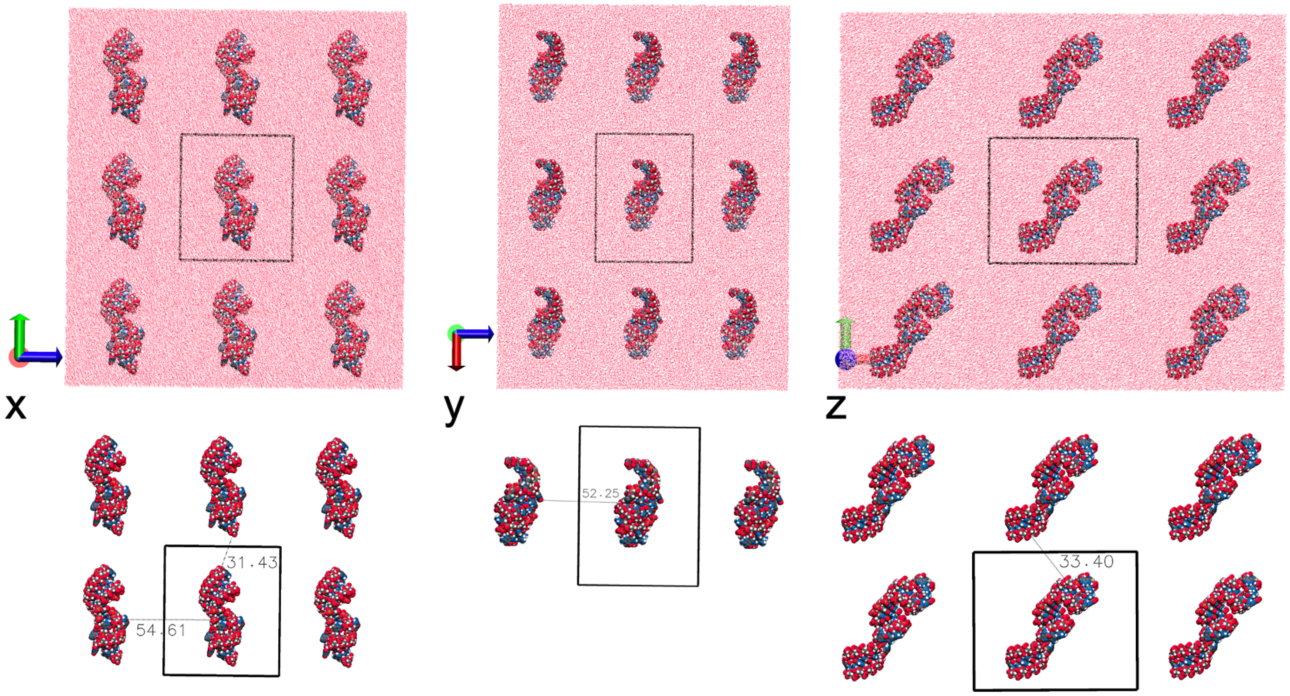


Figure S1. Solvation box built with an initial 15 Å of TIP3P padding from the solute shown after equilibration (black outline) with periodic images in three dimensions (top panel). Dashed lines illustrate the distance between s2m and a mirror image s2m in angstroms (Å) for the representative frame (bottom panel).

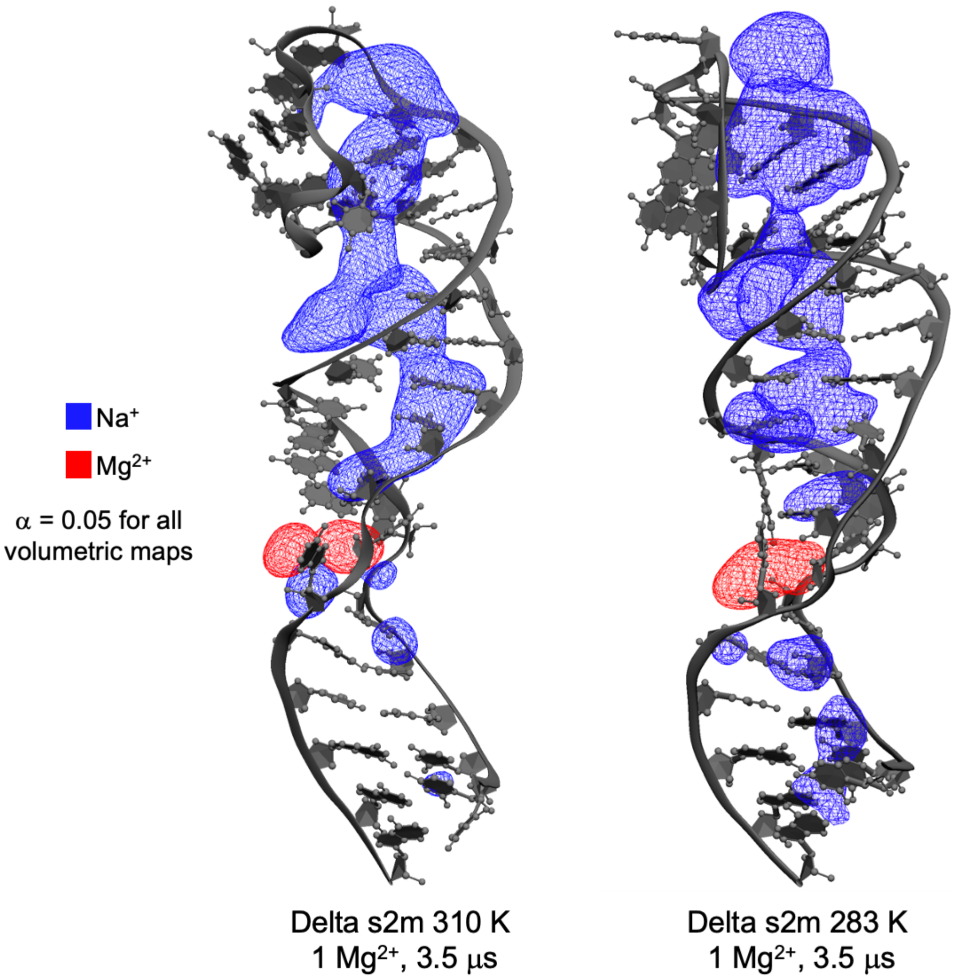


Figure S2. Simulated Delta s2m RNA hairpins at 310 K and 283 K with isosurface maps of the condensed counterion density calculated with a 95% isovalue averaged over 17500 trajectory frames for 3.5 μs.

# Nucleic Heavy Atom Delta s2m 310 K

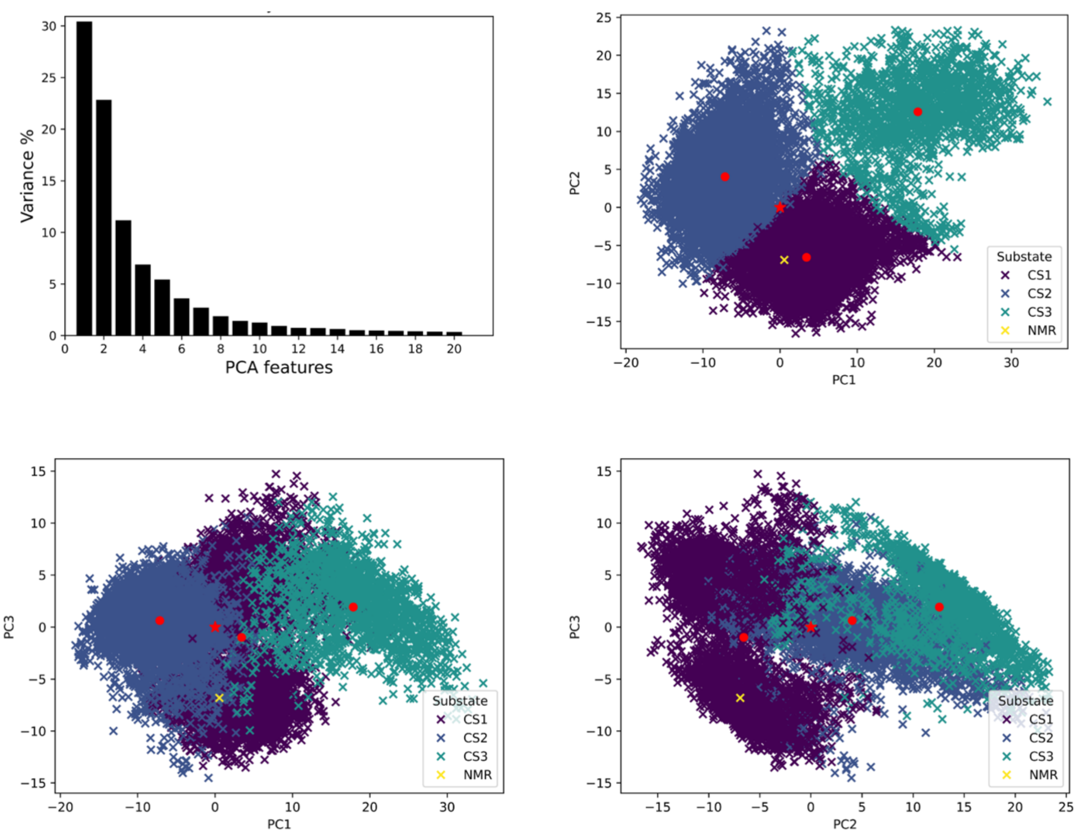


Figure S3A. Scree plot and PC-space projection of all heavy atom coordinates in 310 K Delta s2m. The position of the average structure is given by a red star, CS centroids by red dots, and the starting coordinates by a yellow cross.

# Nucleic Heavy Atom Delta s2m 283 K

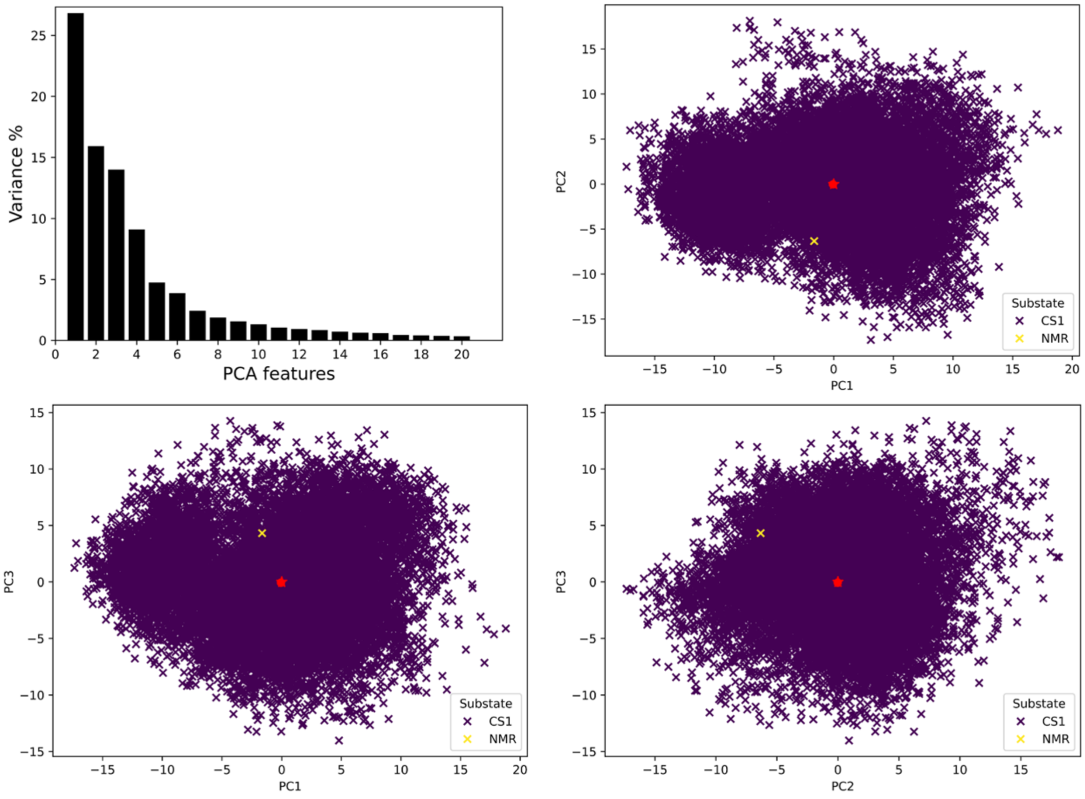


Figure S3B. Scree plot and PC-space projection of all heavy atom coordinates in 283 K Delta s2m. The position of the average structure is given by a red star, CS centroids by red dots, and the starting coordinates by a yellow cross.

# Nucleic Heavy Atom Delta s2m Terminal Loop 310 K

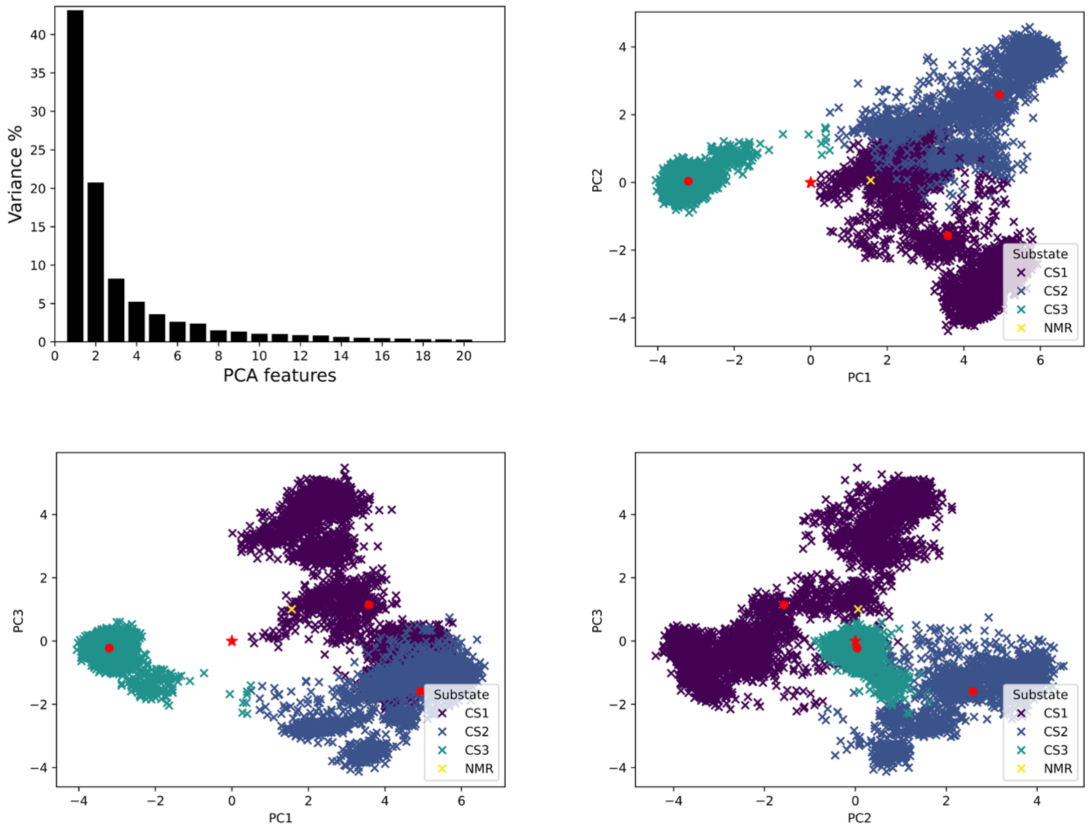


Figure S3C. Scree plot and PC-space projection of all terminal loop heavy atom coordinates in 310 K Delta s2m. The position of the average structure is given by a red star, CS centroids by red dots, and the starting coordinates by a yellow cross.

# Nucleic Heavy Atom Delta s2m Terminal Loop 283 K

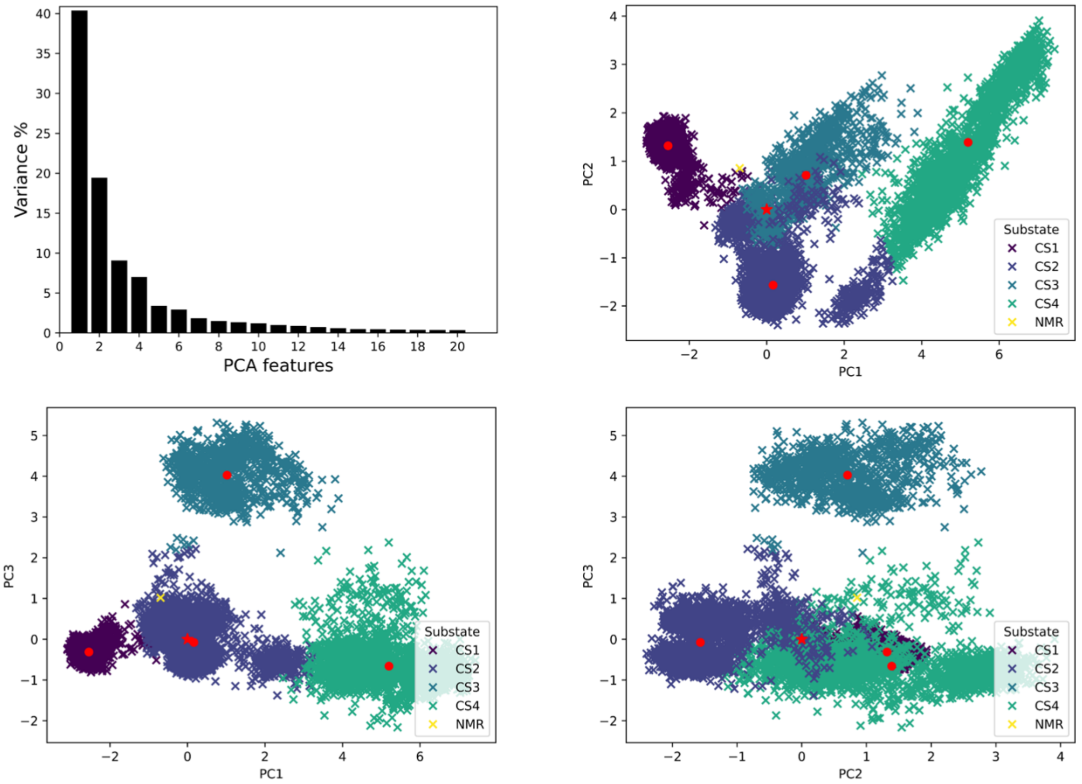


Figure S3D. Scree plot and PC-space projection of all terminal loop heavy atom coordinates in 283 K Delta s2m. The position of the average structure is given by a red star, CS centroids by red dots, and the starting coordinates by a yellow cross.

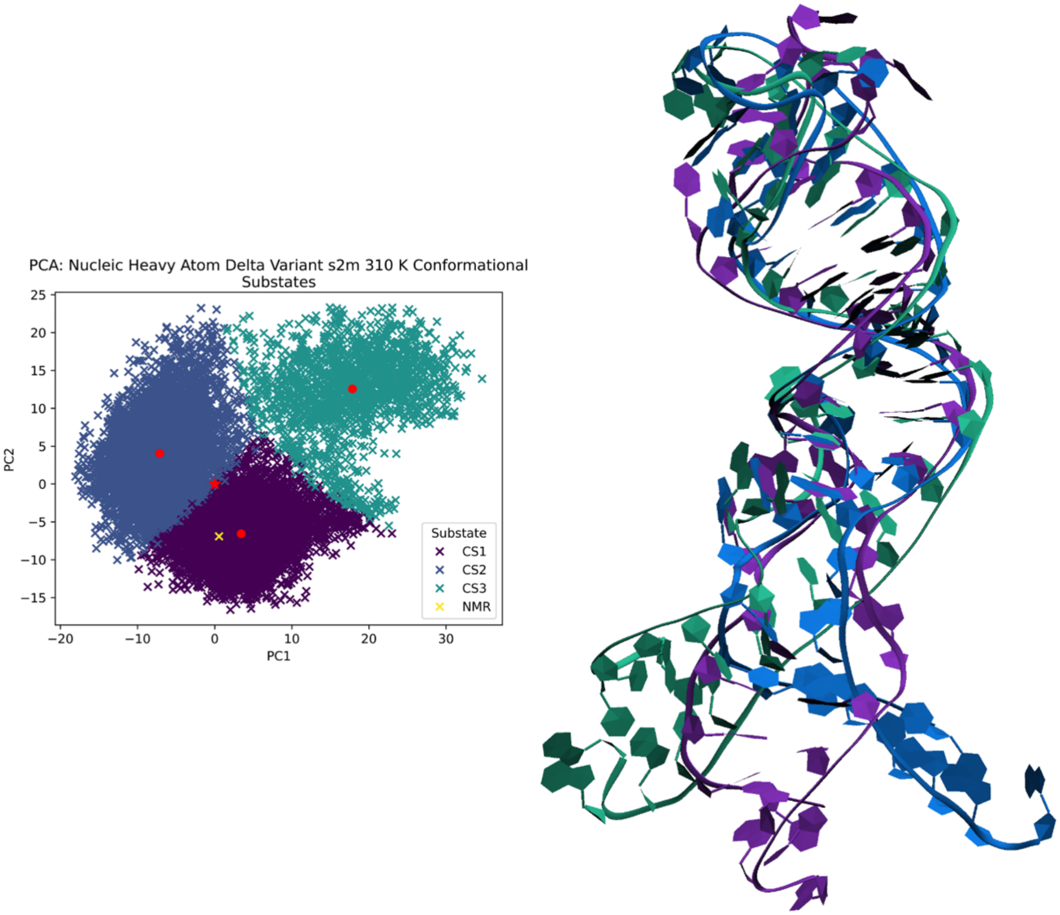


Figure S4. Large magnitude motion in the lower stem of the Delta s2m at 310 K. The position of the average structure is given by a red star, CS centroids by red dots, and the starting coordinates by a yellow cross.

**Table S1. Web3DNA Delta s2m CS1 upper stem helical parameters.**[a]

| | step | Xp | Yp | Zp | XpH | YpH | ZpH | Form |
|---|---|---|---|---|---|---|---|---|
| 1 | CG/CG | -1.02 | 8.42 | 2.78 | -5.84 | 8.25 | 3.00 | A |
| 2 | GA/**U**C | -1.70 | 9.09 | 2.01 | -5.67 | 8.16 | 3.49 | A |
| 3 | AG/C**U** | -2.14 | 7.24 | 2.11 | -6.31 | 6.77 | 5.24 | A |
| 4 | GU/AC | -1.97 | 7.97 | 1.68 | -5.57 | 8.20 | 3.14 | A |
| 5 | UG/CA | -2.41 | 8.1 | 1.03 | -6.78 | 5.91 | 7.03 | |
| 6 | GU/AC | -2.38 | 8.07 | 0.99 | -5.17 | 8.28 | 3.11 | |
| Ideal[b] | AA/UU | -1.18 | 8.35 | 2.65 | -5.07 | 7.39 | 4.71 | A |

[a]Classification of each dinucleotide step in a right-handed nucleic acid structure: A-form helix identified. [b]Ideal A-form helix RNA (polyA-polyU) was generated and analyzed using web3DNA for comparison to the CS1 centroid values. Nucleotides underlined and colored in red are the locations of U15 in Delta s2m.

**Table S2. Web3DNA Delta s2m CS2 upper stem helical parameters.**[a]

| | step | Xp | Yp | Zp | XpH | YpH | ZpH | Form |
|---|---|---|---|---|---|---|---|---|
| 1 | CG/CG | -3.39 | 9.02 | 0.24 | -8.90 | 5.12 | 7.51 | |
| 2 | GA/**U**C | -4.08 | 8.74 | 0.63 | -7.71 | 8.71 | 0.97 | |
| 3 | AG/C**U** | -3.32 | 8.32 | 1.51 | -7.86 | 5.68 | 6.22 | A |
| 4 | GU/AC | -1.62 | 8.72 | 1.84 | -5.09 | 8.21 | 3.48 | A |
| 5 | UG/CA | -1.52 | 8.53 | 2.31 | -5.50 | 7.59 | 4.53 | A |
| 6 | GU/AC | -1.86 | 8.41 | 2.60 | -6.20 | 7.09 | 5.19 | |
| Ideal[b] | AA/UU | -1.18 | 8.35 | 2.65 | -5.07 | 7.39 | 4.71 | A |

[a]Classification of each dinucleotide step in a right-handed nucleic acid structure: A-form helix identified. [b]Ideal A-form helix RNA (polyA-polyU) was generated and analyzed using web3DNA for comparison to the CS1 centroid values. Nucleotides underlined and colored in red are the locations of U15 in Delta s2m.

**Table S3. Web3DNA Delta s2m CS3 upper stem helical parameters.**[a]

| | step | Xp | Yp | Zp | XpH | YpH | ZpH | Form |
|---|---|---|---|---|---|---|---|---|
| 1 | CC/GU | -1.17 | 9.19 | 0.81 | -1.77 | 8.79 | 3.04 | |
| 2 | CA/UG | -1.27 | 8.53 | 2.16 | -4.57 | 6.58 | 5.83 | A |
| 3 | AC/GU | -1.95 | 8.47 | 2.02 | -5.72 | 7.43 | 4.54 | A |
| 4 | C**U**/AG | -2.10 | 8.55 | 2.39 | -8.01 | 6.81 | 5.55 | A |
| 5 | **U**C/GA | -1.50 | 8.62 | 2.05 | -2.67 | 8.77 | 1.14 | A |
| 6 | CG/CG | -3.38 | 8.13 | 2.52 | -6.87 | 5.88 | 5.54 | |
| Ideal[b] | AA/UU | -1.18 | 8.35 | 2.65 | -5.07 | 7.39 | 4.71 | A |

[a]Classification of each dinucleotide step in a right-handed nucleic acid structure: A-form helix identified. [b]Ideal A-form helix RNA (polyA-polyU) was generated and analyzed using web3DNA for comparison to the CS1 centroid values. Nucleotides uunderlined and colored in red are the locations of U15 in Delta s2m.

**Table S4. Web3DNA SARS-CoV-2 s2m CS1 upper stem helical parameters.**[a]

| | step | Xp | Yp | Zp | XpH | YpH | ZpH | Form |
|---|---|---|---|---|---|---|---|---|
| 1 | CG/CG | -2.44 | 9.09 | 1.44 | -3.44 | 9.25 | 0.86 | |
| 2 | GA/**G**C | -2.21 | 7.24 | 2.55 | -16.86 | 0.36 | 7.75 | |
| 3 | AG/C**G** | -3.07 | 7.97 | 1.74 | -3.62 | 7.83 | 2.58 | |
| 4 | GU/AC | -2.63 | 8.1 | 2.58 | -7.12 | 7.33 | 4.26 | A |
| 5 | UG/CA | -2.11 | 8.07 | 2.78 | -5.85 | 4.95 | 6.88 | A |
| 6 | GU/AC | -1.33 | 7.77 | 3.51 | -4.83 | 7.49 | 3.97 | |
| Ideal[b] | AA/UU | -1.18 | 8.35 | 2.65 | -5.07 | 7.39 | 4.71 | A |

[a]Classification of each dinucleotide step in a right-handed nucleic acid structure: A-form helix identified. [b]Ideal A-form helix RNA (polyA-polyU) was generated and analyzed using web3DNA for comparison to the CS1 centroid values. Nucleotides underlined and colored in red are the locations of U15 in Delta s2m.

**Table S5. Web3DNA Local base-pair step parameters.**

| | step | Shift (Å) | Slide (Å) | Rise (Å) | Tilt (°) | Roll (°) | Twist (°) |
|---|---|---|---|---|---|---|---|
| SARS-CoV-2 | AG/CG | -0.40 | -0.73 | 3.41 | -10.0 | 4.3 | 61.0 |
| Delta | AG/CU | -0.70 | -2.38 | 3.70 | 5.81 | 10.4 | 21.9 |
| Ideal[a] | AA/UU | -0.08 | -1.48 | 3.30 | -0.44 | 8.64 | 31.5 |

[a]Ideal A-form helix RNA (polyA-polyU) was generated and analyzed using web3DNA for comparison to the CS1 centroid values. Nucleotides colored in red are the locations of G15 in SARS-CoV-2 s2m or U15 in Delta s2m.

**Table S6. Web3DNA Local base-pair helical parameters.**

| | step | X-disp (Å) | Y-disp (Å) | h-Rise (Å) | Incl. (°) | Tip (°) | h-Twist (°) |
|---|---|---|---|---|---|---|---|
| SARS-CoV-2 | AG/CG | -0.93 | -0.10 | 3.38 | 4.26 | 9.77 | 61.9 |
| Delta | AG/CU | -8.50 | 3.28 | 2.11 | 25.1 | -14.0 | 24.8 |
| Ideal[a] | AA/UU | -4.05 | 0.07 | 2.81 | 15.5 | 0.79 | 32.7 |

[a]Ideal A-form helix RNA (polyA-polyU) was generated and analyzed using web3DNA for comparison to the CS1 centroid values. Nucleotides underlined and colored in red are the locations of G15 in SARS-CoV-2 s2m or U15 in Delta s2m.

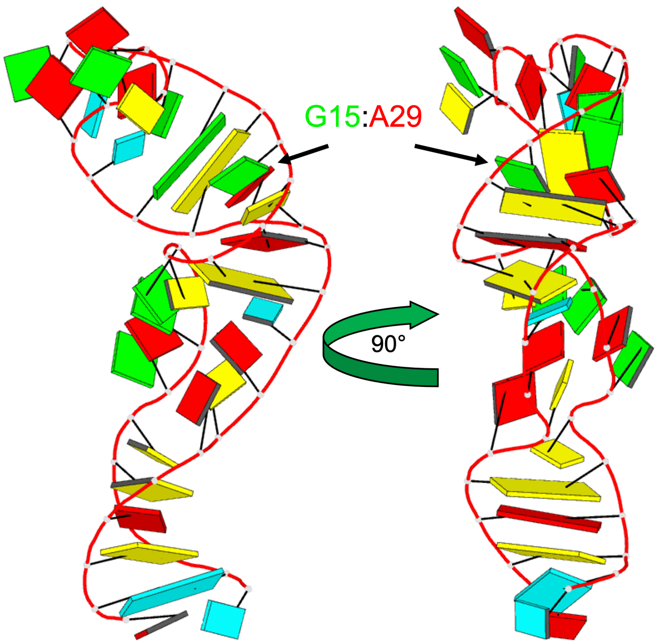


Figure S5. SARS-CoV-2 s2m noncanonical base step changes the helical direction inducing an overall kinked shape. CS1 is representative of the global shape observed in each SARS-CoV- 2 terminal loop CS.

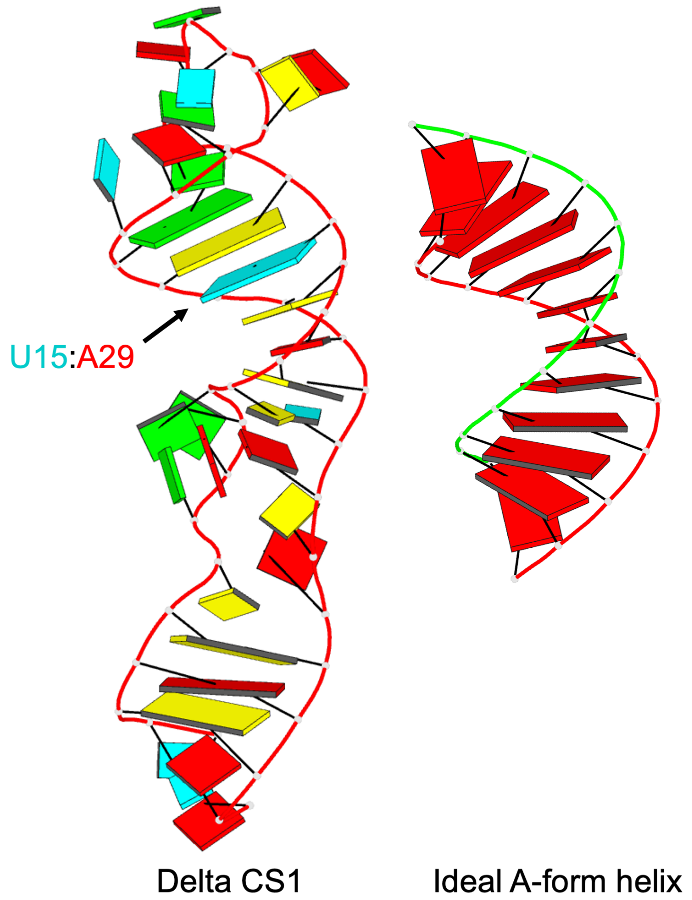


Figure S6. Delta canonical base step allows for standard helical shape aligned with A-form helical parameters and does not induce a kinked shape. CS1 is representative of the global shape observed in each Delta terminal loop CS.

# Nucleic Heavy Atom Delta s2m Terminal Loop 310 K
# CS1 Tier-2

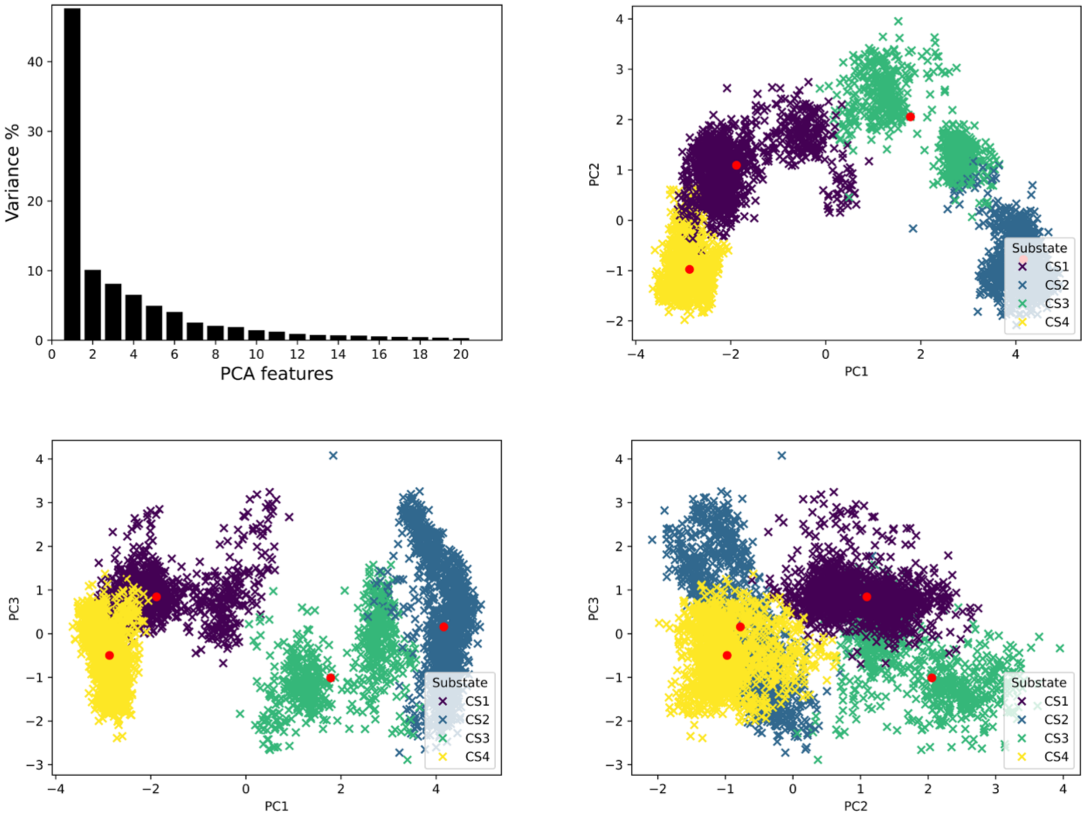


Figure S7A. Scree plot and PC-space projection of all terminal loop heavy atom coordinates in CS1 of the 310 K Delta s2m, yielding four Tier-2 CS. The position of the average structure is given by a red star and Tier-2 CS centroids by red dots. CS1 corresponds to the Tier-2 CS containing the Tier-1 centroid structure, while other CS are numbered in approximately chronological order.

# Nucleic Heavy Atom Delta s2m Terminal Loop 310 K
# CS2 Tier-2

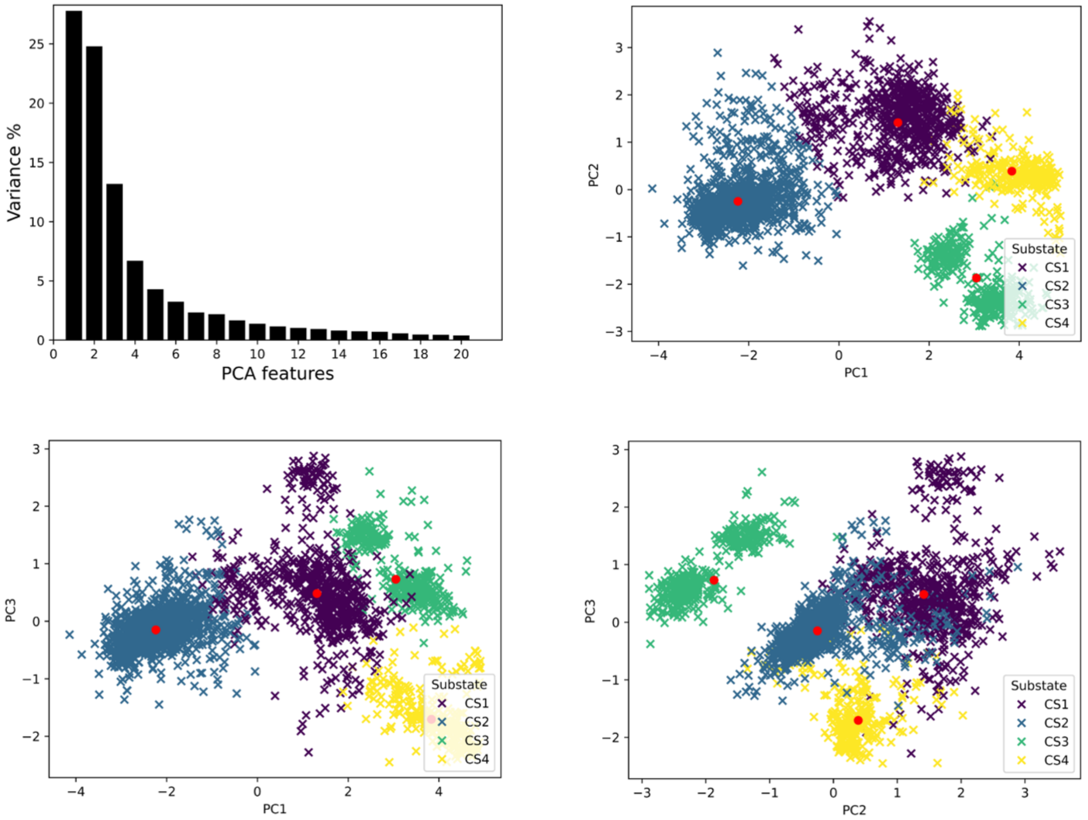


Figure S7B. Scree plot and PC-space projection of all terminal loop heavy atom coordinates in CS2 of the 310 K Delta s2m, yielding four Tier-2 CS. The position of the average structure is given by a red star and Tier-2 CS centroids by red dots. CS1 corresponds to the Tier-2 CS containing the Tier-1 centroid structure, while other CS are numbered in approximately chronological order.

# Nucleic Heavy Atom Delta s2m Terminal Loop 310 K
# CS3 Tier-2

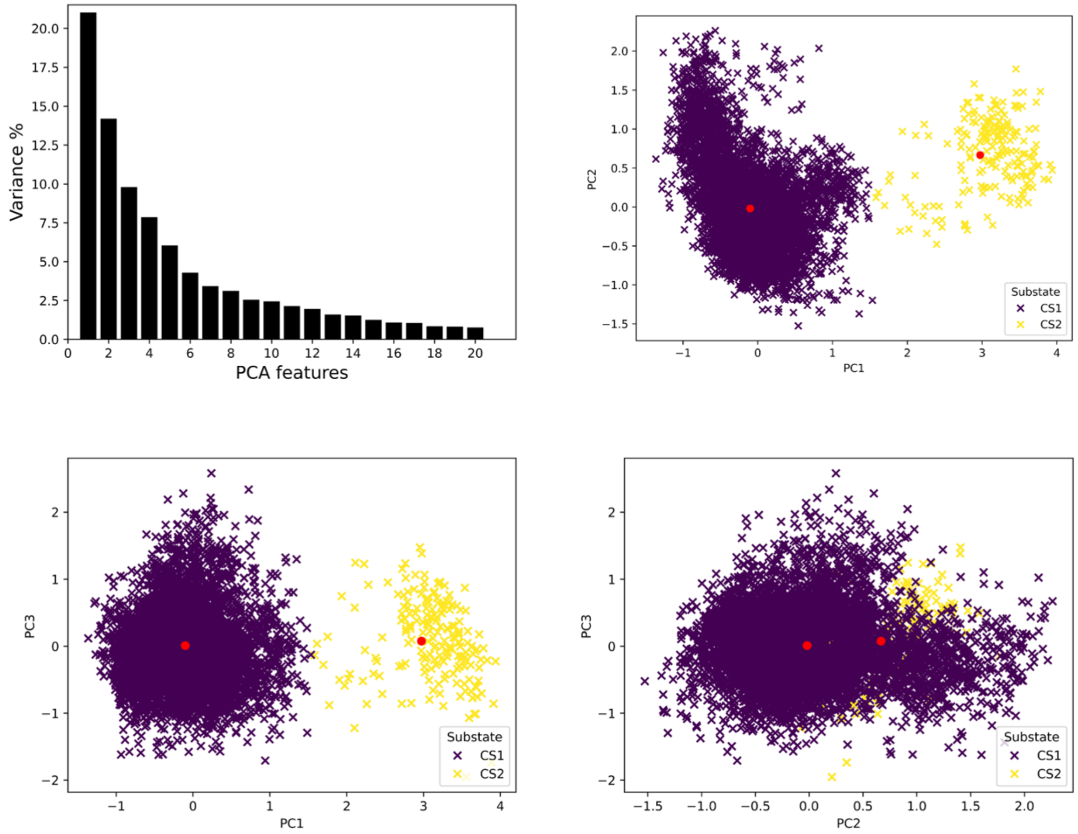


Figure S7C. Scree plot and PC-space projection of all terminal loop heavy atom coordinates in CS3 of the 310 K Delta s2m, yielding two Tier-2 CS. The position of the average structure is given by a red star and Tier-2 CS centroids by red dots. CS1 corresponds to the Tier-2 CS containing the Tier-1 centroid structure, while other CS are numbered in approximately chronological order.

# Nucleic Heavy Atom Delta s2m Terminal Loop 283 K
# CS1 Tier-2

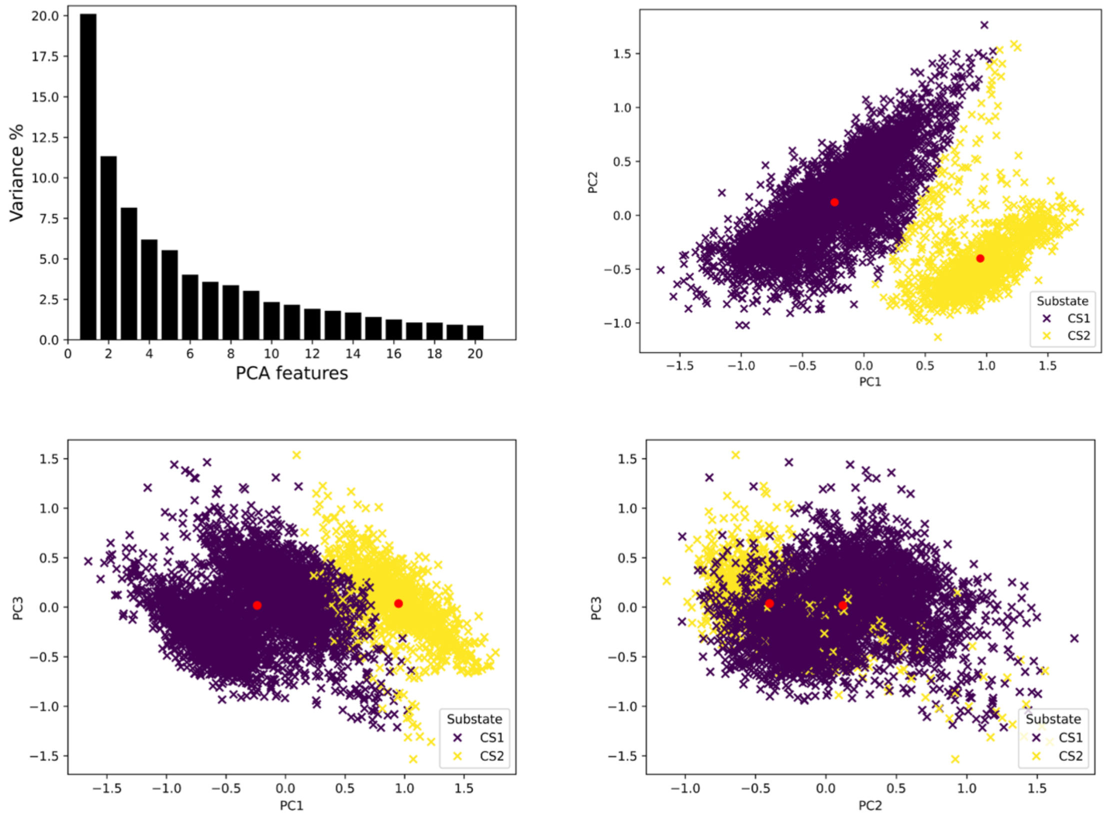


Figure S7D. Scree plot and PC-space projection of all terminal loop heavy atom coordinates in CS1 of the 283 K Delta s2m, yielding two Tier-2 CS. The position of the average structure is given by a red star and Tier-2 CS centroids by red dots. CS1 corresponds to the Tier-2 CS containing the Tier-1 centroid structure, while other CS are numbered in approximately chronological order.

# Nucleic Heavy Atom Delta s2m Terminal Loop 283 K
# CS2 Tier-2

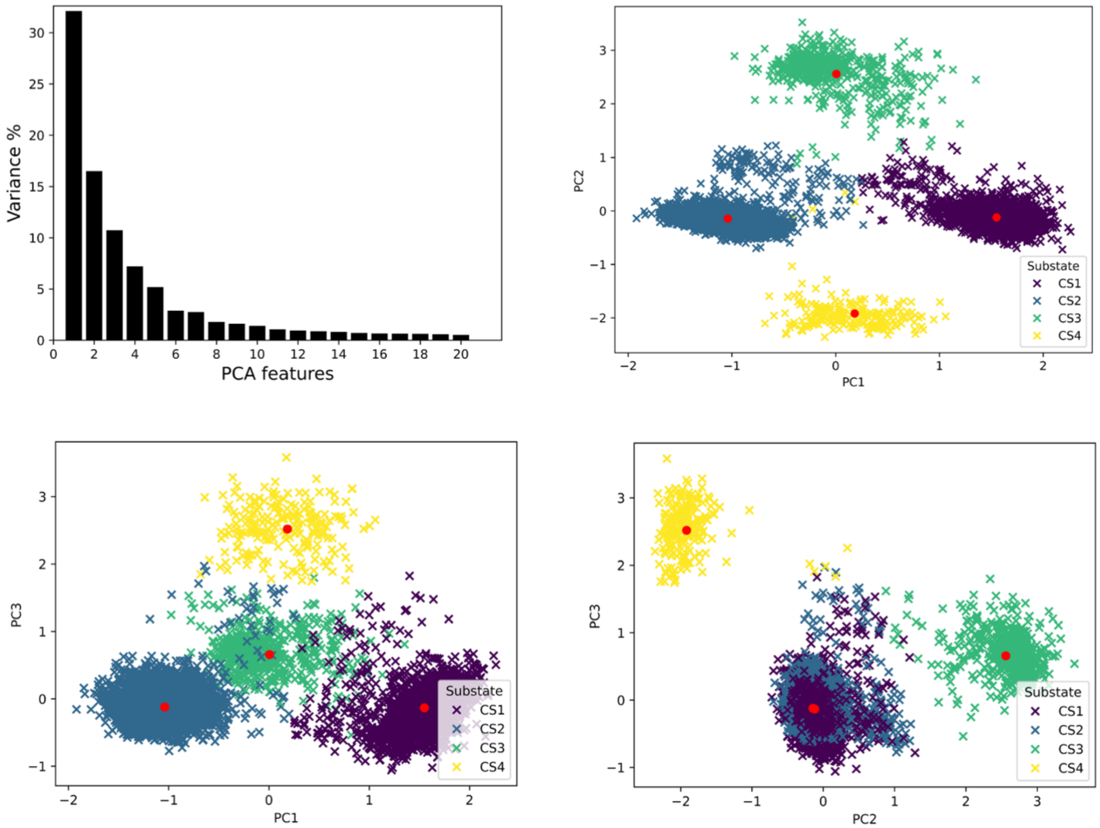


Figure S7E. Scree plot and PC-space projection of all terminal loop heavy atom coordinates in CS2 of the 283 K Delta s2m, yielding four Tier-2 CS. The position of the average structure is given by a red star and Tier-2 CS centroids by red dots. CS1 corresponds to the Tier-2 CS containing the Tier-1 centroid structure, while other CS are numbered in approximately chronological order.

# Nucleic Heavy Atom Delta s2m Terminal Loop 283 K
# CS3 Tier-2

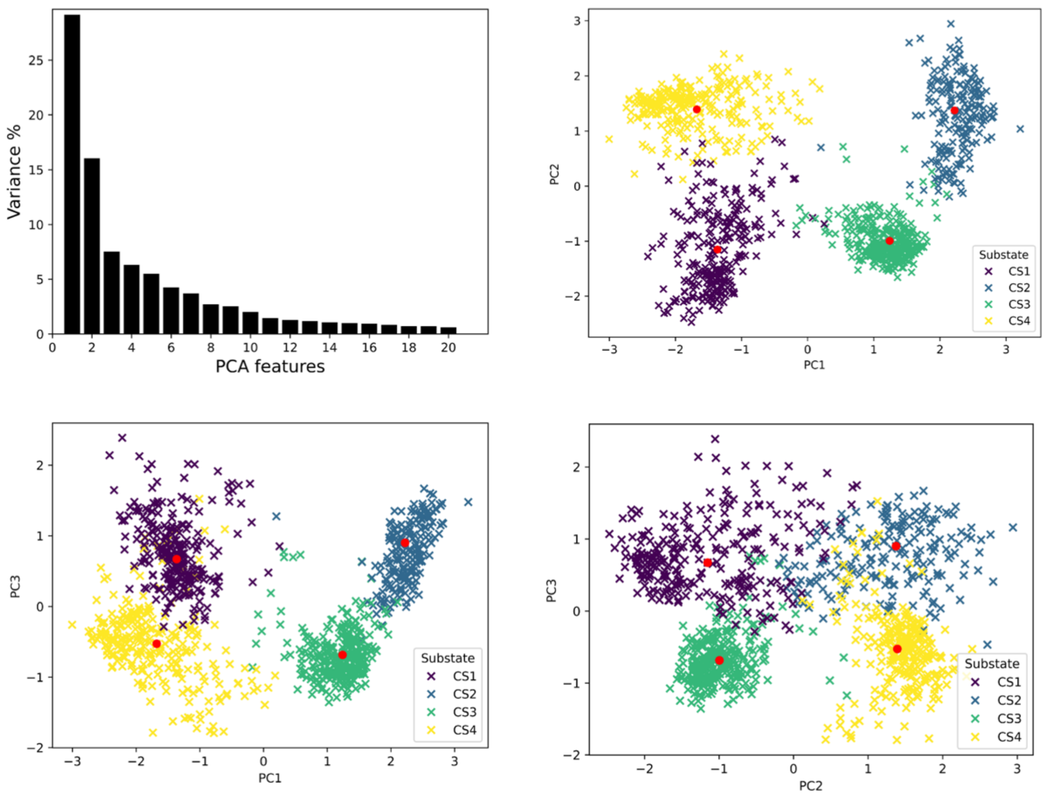


Figure S7F. Scree plot and PC-space projection of all terminal loop heavy atom coordinates in CS3 of the 283 K Delta s2m, yielding four Tier-2 CS. The position of the average structure is given by a red star and Tier-2 CS centroids by red dots. CS1 corresponds to the Tier-2 CS containing the Tier-1 centroid structure, while other CS are numbered in approximately chronological order.

# Nucleic Heavy Atom Delta s2m Terminal Loop 283 K
# CS4 Tier-2

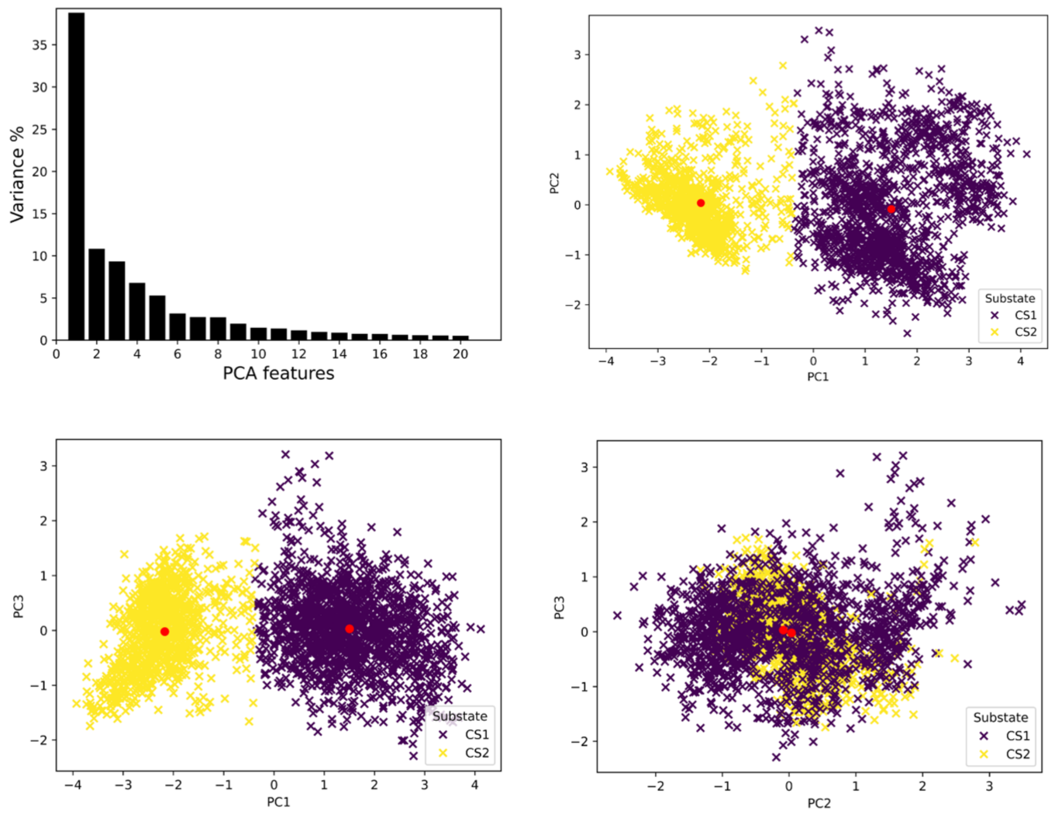


Figure S7G. Scree plot and PC-space projection of all terminal loop heavy atom coordinates in CS4 of the 283 K Delta s2m, yielding two Tier-2 CS. The position of the average structure is given by a red star and Tier-2 CS centroids by red dots. CS1 corresponds to the Tier-2 CS containing the Tier-1 centroid structure, while other CS are numbered in approximately chronological order.

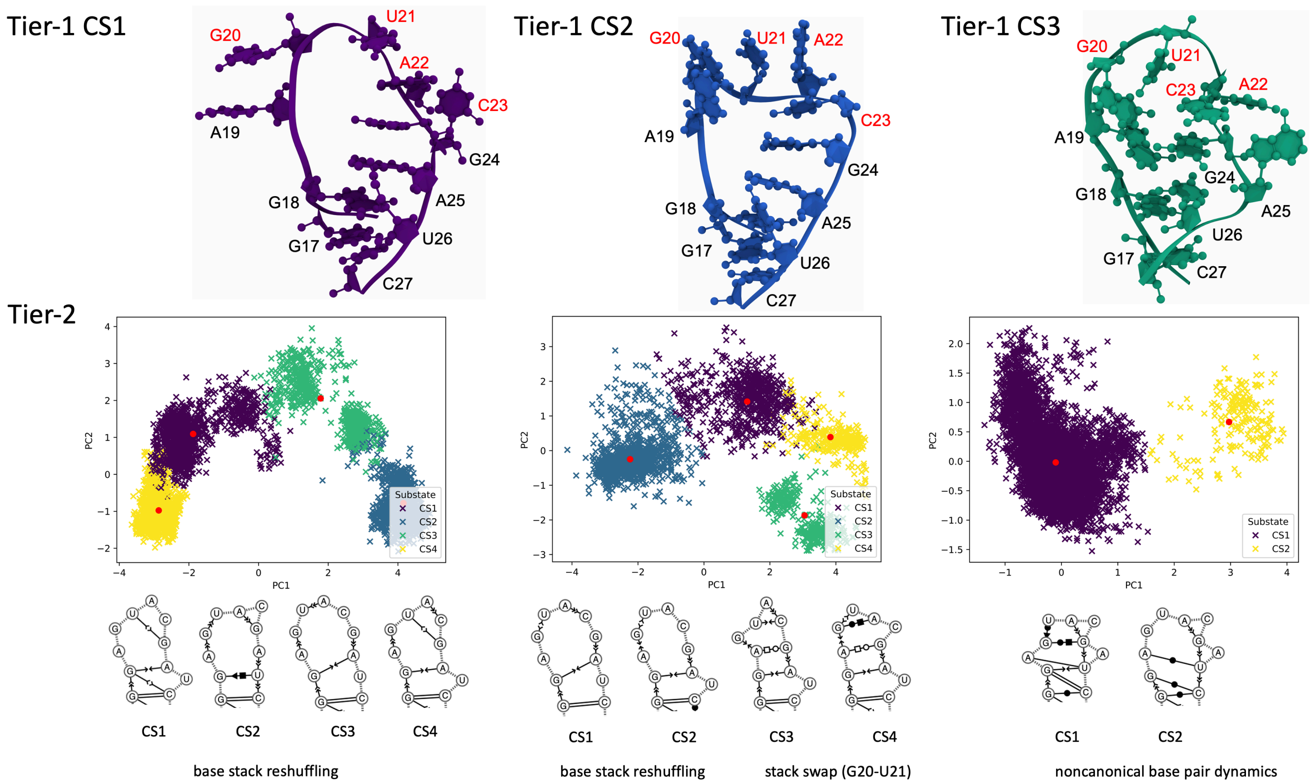


Figure S7H. Tier-2 PCA and centroid secondary structures within each Tier-1 CS from 310 K terminal loop PCA. The top row depicts each Tier-1 CS centroid, with the frames of the corresponding CS re-partitioned into new Tier-2 CS. Tier-2 dynamics are summarized below the centroid secondary structures.

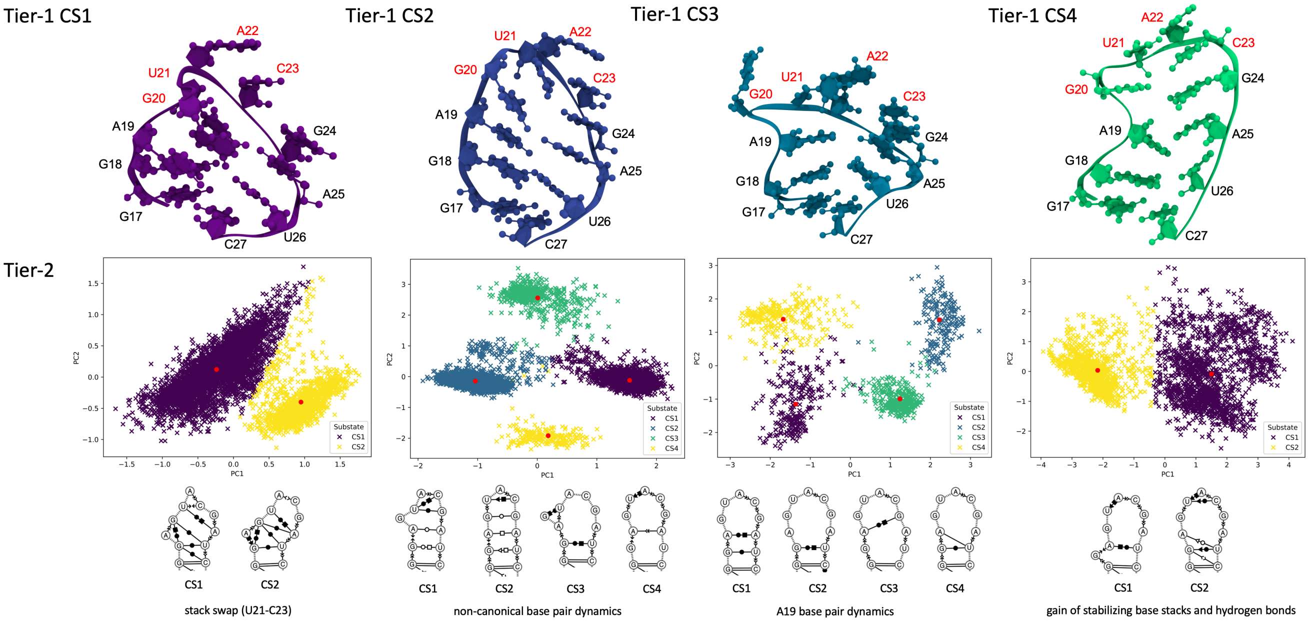


Figure S7I. Tier-2 PCA and centroid secondary structures within each Tier-1 CS from 283 K terminal loop PCA. The top row depicts each Tier-1 CS centroid, with the frames of the corresponding CS re-partitioned into new Tier-2 CS. Tier-2 dynamics are summarized below the centroid secondary structures.

**Methods**

**Estimation of Absolute Entropy**. A complete account of the quasiharmonic approximation can be found in the original articles by Andricioaei and Karplus[1] and Schlitter.[2] Practically, by finding the eigenvalues of the mass-weighted covariance matrix $\mathbf{C}'$,

$$\mathbf{C}' = \mathbf{M}^{1/2}\mathbf{C}\mathbf{M}^{1/2}$$

for which **M** is the block matrix of atomic masses on the diagonal and elsewhere **0**, one may determine a set of quasiharmonic frequencies ω from the eigenvalues λ of $\mathbf{C}'$ by the relation

$$\omega_i = \sqrt{\frac{kT}{\lambda_i}}$$

which, by the quantum harmonic oscillator partition function, yields the absolute entropy

$$S_{\text{ho}} = k\sum_{i}^{3n-6}\frac{\hbar\omega_i/kT}{e^{\hbar\omega_i/kT}-1} - \ln\left(1 - e^{-\hbar\omega_i/kT}\right)$$

over the $3n$-6 vibrational modes with nonzero eigenvalues, $n$ the number of atoms. Noting the dependence on $T$, we used our 310 K Delta s2m simulation to compare entropy with our previously reported values,[3] which were derived from simulations of the SARS-CoV s2m and SARS-CoV-2 s2m performed at 310 K.

**References**


(1) Andricioaei, I.; Karplus, M. On the Calculation of Entropy from Covariance Matrices of the Atomic Fluctuations. *Journal of Chemical Physics* **2001**, *115* (14), 6289–6292. https://doi.org/10.1063/1.1401821.

(2) Schlitter, J. Estimation of Absolute and Relative Entropies of Macromolecules Using the Covariance Matrix. *Chem Phys Lett* **1993**, *215* (6), 617–621. https://doi.org/10.1016/0009-2614(93)89366-P.

(3) Kensinger, A. H.; Makowski, J. A.; Pellegrene, K. A.; Imperatore, J. A.; Cunningham, C. L.; Frye, C. J.; Lackey, P. E.; Mihailescu, M. R.; Evanseck, J. D. Structural, Dynamical, and Entropic Differences between SARS-CoV and SARS-CoV-2 S2m Elements Using Molecular Dynamics Simulations. *ACS Physical Chemistry Au* **2022**. https://doi.org/10.1021/acsphyschemau.2c00032.
